# Striatal synaptic bioenergetic and autophagic decline in premotor experimental parkinsonism

**DOI:** 10.1093/brain/awac087

**Published:** 2022-03-04

**Authors:** Leyre Merino-Galán, Haritz Jimenez-Urbieta, Marta Zamarbide, Tatiana Rodríguez-Chinchilla, Arantzazu Belloso-Iguerategui, Enrique Santamaria, Joaquín Fernández-Irigoyen, Ana Aiastui, Evelyne Doudnikoff, Erwan Bézard, Alberto Ouro, Shira Knafo, Belén Gago, Ana Quiroga-Varela, María Cruz Rodríguez-Oroz

**Affiliations:** Neuroscience Program, Center for Applied Medical Research (CIMA), Universidad de Navarra, 31008 Pamplona, Spain; Neuroscience Department, University of the Basque Country (UPV/EHU), 48940 Leioa, Spain; Cell Culture Platform, Biodonostia Health Research Institute, San Sebastian, 20014 Donostia, Spain; Neuroscience Program, Center for Applied Medical Research (CIMA), Universidad de Navarra, 31008 Pamplona, Spain; Neuroscience Program, Center for Applied Medical Research (CIMA), Universidad de Navarra, 31008 Pamplona, Spain; Neuroscience Program, Center for Applied Medical Research (CIMA), Universidad de Navarra, 31008 Pamplona, Spain; Clinical Neuroproteomics Unit, Proteomics Platform, Proteored-ISCIII, Navarrabiomed, Complejo Hospitalario de Navarra (CHN), Universidad Pública de Navarra (UPNA), 31008 Pamplona, Spain; Navarra Institute for Health Research (IdiSNA), Pamplona, Spain; Clinical Neuroproteomics Unit, Proteomics Platform, Proteored-ISCIII, Navarrabiomed, Complejo Hospitalario de Navarra (CHN), Universidad Pública de Navarra (UPNA), 31008 Pamplona, Spain; Navarra Institute for Health Research (IdiSNA), Pamplona, Spain; Cell Culture Platform, Biodonostia Health Research Institute, San Sebastian, 20014 Donostia, Spain; CNRS, Institut des Maladies Neurodégénératives, UMR 5293, 33076 Bordeaux, France; CNRS, Institut des Maladies Neurodégénératives, UMR 5293, 33076 Bordeaux, France; Clinical Neurosciences Research Laboratories, Health Research Institute of Santiago de Compostela (IDIS), 15706 Santiago de Compostela, Spain; Department of Physiology and Cell Biology, Faculty of Health Sciences, The National Institute for Biotechnology in the Negev, and The Zlotowski Center for Neuroscience, Ben-Gurion University of the Negev, 8410501 Beer-Sheva, Israel; Instituto Biofisika (UPV/EHU, CSIC), University of the Basque Country, Basque Foundation for Science, IKERBASQUE, 48940 Leioa, Spain; Faculty of Medicine, Instituto de Investigación Biomédica de Málaga, Universidad de Málaga, 29016 Málaga, Spain; Neuroscience Program, Center for Applied Medical Research (CIMA), Universidad de Navarra, 31008 Pamplona, Spain; Navarra Institute for Health Research (IdiSNA), Pamplona, Spain; Neuroscience Program, Center for Applied Medical Research (CIMA), Universidad de Navarra, 31008 Pamplona, Spain; Navarra Institute for Health Research (IdiSNA), Pamplona, Spain; Neurology Department, Clínica Universidad de Navarra (CUN), 31008 Pamplona, Spain

**Keywords:** α-synuclein, striatum, Parkinson’s disease, synapse, mitochondria

## Abstract

Synaptic impairment might precede neuronal degeneration in Parkinson’s disease. However, the intimate mechanisms altering synaptic function by the accumulation of presynaptic α-synuclein in striatal dopaminergic terminals before dopaminergic death occurs, have not been elucidated. Our aim is to unravel the sequence of synaptic functional and structural changes preceding symptomatic dopaminergic cell death. As such, we evaluated the temporal sequence of functional and structural changes at striatal synapses before parkinsonian motor features appear in a rat model of progressive dopaminergic death induced by overexpression of the human mutated A53T α-synuclein in the substantia nigra pars compacta, a protein transported to these synapses. Sequential window acquisition of all theoretical mass spectra proteomics identified deregulated proteins involved first in energy metabolism and later, in vesicle cycling and autophagy. After protein deregulation and when α-synuclein accumulated at striatal synapses, alterations to mitochondrial bioenergetics were observed using a Seahorse XF96 analyser. Sustained dysfunctional mitochondrial bioenergetics was followed by a decrease in the number of dopaminergic terminals, morphological and ultrastructural alterations, and an abnormal accumulation of autophagic/endocytic vesicles inside the remaining dopaminergic fibres was evident by electron microscopy. The total mitochondrial population remained unchanged whereas the number of ultrastructurally damaged mitochondria increases as the pathological process evolved. We also observed ultrastructural signs of plasticity within glutamatergic synapses before the expression of motor abnormalities, such as a reduction in axospinous synapses and an increase in perforated postsynaptic densities. Overall, we found that a synaptic energetic failure and accumulation of dysfunctional organelles occur sequentially at the dopaminergic terminals as the earliest events preceding structural changes and cell death. We also identify key proteins involved in these earliest functional abnormalities that may be modulated and serve as therapeutic targets to counterbalance the degeneration of dopaminergic cells to delay or prevent the development of Parkinson’s disease.

## Teaser

Merino-Galan *et al.* evaluate the temporal sequence of functional and ultrastructural changes at striatal synapses prior to the emergence of parkinsonian motor features. They identify key proteins involved in these functional abnormalities that could be targeted to help delay or prevent dopaminergic degeneration.

**See Aleph Prieto and Cotman (https://doi.org/10.1093/brain/awac191) for a scientific commentary on this article.**

## Introduction

Parkinson’s disease is a progressive neurodegenerative disorder characterized by the degeneration of dopaminergic neurons in the substantia nigra pars compacta (SN_pc_), and the loss of dopaminergic innervation and dopamine in the striatum. The accumulation of intracellular inclusions (Lewy bodies), in which α-synuclein (α-syn) is the main protein is the pathological hallmark of Parkinson’s disease.^1,2^ Neurodegeneration in Parkinson’s disease begins years beforehand, often in conjunction with a variety of non-motor symptoms.^3^ This long latent phase of the disease, termed premotor Parkinson’s disease, represents an opportunity to study the initial pathophysiological changes in Parkinson’s disease with the ultimate goal of developing therapies able to delay or prevent the degeneration of dopaminergic neurons.

The synapse is the most physiologically active neuronal compartment, and it relies on adequate metabolic and mitochondrial activity to provide the ATP that powers synaptic vesicle cycling and to sustain repeated neurotransmitter release.^4^ Dopaminergic neurons of the SN_pc_ have large axonal arbourizations, containing vast numbers of synaptic terminals that demand a high-energy supply to sustain their intrinsically elevated electrical excitability.^5,6^ This complex axonal arbour means they are controlled by a highly regulated energy budget, which makes them particularly susceptible to factors that might induce cell stress. In this sense, stressors that perturb energy production such as α-syn aggregation may drive energy demand beyond that of supply, consequently inducing cell death.^5,6^ Several lines of evidence point to synaptic failure as the first step in the dopaminergic degeneration associated with Parkinson’s disease, as the loss of striatal dopaminergic terminals seems to precede neuronal loss in the SN_pc_.^7–9^ Thus, abnormal interactions of aggregated α-syn in dopaminergic synapses could be the molecular basis underlying the functional defects at synapses that compromise neuronal communication^10–14^ and that lead to neuronal degeneration.

On the other hand, the glutamatergic system also plays a prominent role in the striatal function as excitatory striatal asymmetric synapses (ASs) account for about 80% of all striatal synapses.^15,16^ Dopamine modulates the synaptic plasticity of these synapses being their function altered in Parkinson’s disease. Notably, in models of stablished dopaminergic degeneration with motor abnormalities, surviving dopaminergic neurons have been associated with enhanced synaptic strength in corticostriatal glutamatergic circuits.^17–19^ This has been interpreted as a compensatory plastic response to the changes in dopamine homeostasis in an attempt to preserve synaptic dopamine availability.^20–23^ However, it is not known whether the earliest functional and structural alterations in the nigrostriatal dopaminergic synapses might involve modifications in striatal glutamatergic architecture, which could lead to changes in striatal synaptic plasticity even before significant dopaminergic cell loss occurs.^18,19,23,24^

Although synaptic dysfunction seems to be an early pathological event in Parkinson’s disease, preceding neuronal demise, the intimate mechanisms driving these early synaptic changes and the plastic mechanisms that might stabilize the premotor phase remain elusive. This is because most research in this field has been conducted both in Parkinson’s disease patients and in animal models once dopaminergic neurodegeneration produces a phenotypic effect (i.e. once >50% cell loss has occurred), as occurs at Parkinson’s disease diagnosis.^18,23,25–28^ As such, the early changes at the striatal dopaminergic synapse have been poorly characterized. Better understanding the early synaptic events triggered by α-syn accumulation, before the onset of dopaminergic cell death and motor manifestations and, yet once the underlying pathological processes have commenced, could help identify therapeutic strategies that target the synaptic machinery of selective and vulnerable synapses, thereby halting Parkinson’s disease progression.

To address these issues, we set out to study the earliest temporal sequence of functional and structural changes at striatal synapses associated with α-syn overexpression and dopaminergic degeneration. As such, we used an experimental model of progressive parkinsonism in rats that are induced by viral vector-mediated overexpression of A53T mutated human α-syn (hα-syn) in the SN_pc_.

## Materials and methods

### Animal model

Adult male Sprague-Dawley rats (300 g, Charles River) were housed in pairs under standard conditions (70% humidity, 22°C, regular 12-h light/dark cycle) and with *ad libitum* access to food and water. Adeno-associated viral vectors (AAV2/9-CMV-WPRE) inducing the overexpression of either A53T mutated hα-syn (8 × 10^12^ genomic particles/ml) or the empty vector (EVV: 2 × 10^13^ genomic particles/ml) were obtained from the University of Bordeaux (France). A cross-sectional study was performed on animals receiving either hα-syn or EVV at different time points post-inoculation: 24 and 72 h, or 1, 2 and 4 weeks. Rats were anaesthetized with isoflurane in oxygen-enriched air (1–2%) and placed in a stereotactic head frame (Stoelting). The corresponding AAVs were injected bilaterally into the SN_pc_ (1 μl per site, 0.2 μl/min) as described previously.^29,30^ The coordinates from Bregma were: (i) anteroposterior −4.9, lateral ±2.2 and ventral −7.7 mm; and (ii) anteroposterior −5.4, lateral ±2.0 and ventral −7.7 mm.^31^ The injection needle was left in place for 2 min before being slowly retracting it from the brain. All the experimental procedures were approved by the animal research committees of the Biodonostia HRI (CEEA16/11) and CIMA-Universidad de Navarra (107-17), and they were carried out in strict accordance with the guidelines of the Spanish Government (RD53/2013) and the European Union Council Directive (2010/63/EU) on the protection of animals used for scientific purposes. All efforts were made to minimize animal suffering and to reduce the number of animals used.

### Behavioural tests

For *in vivo* monitoring of motor activity, the adjusting stepping test was carried out at baseline, and 24 h, 72 h, 1, 2 and 4 weeks post-inoculation, and the open field test at 1, 2 and 4 weeks post-inoculation The average number of adjusting steps with each forepaw in both directions (adduction and abduction) was analysed,^29,30^ as well as the total distance travelled (cm) and the total velocity (cm/s) for the open field test. For more details see the Supplementary material.

### Immunohistochemistry for hα-syn, TH and the dopamine transporter

Animals (*n* = 4/group at 24 h, 72 h, 1, 2 and 4 weeks post-inoculation) were perfused transcardially with 4% paraformaldehyde—0.2% glutaraldehyde in phosphate buffer (PB, pH 7.4). Brains were removed, post-fixed overnight and then transferred to a cryoprotective solution (pH 7.4) before freeze–thawing in isopentane. Serial coronal vibratome sections (50-µm thick, VT1000S, Leica Microsystems) were collected. Immunohistochemistry was performed on free-floating coronal sections containing the striatum and SN_pc_ to evaluate tyrosine hydroxylase (TH), dopamine transporter and hα-syn expression as described previously.^29,30^ Stereological quantification of TH^+^ neurons and densitometric analysis for hα-syn immunoreactivity was performed in the SN_pc._ The densitometry for TH, hα-syn and dopamine transporter expression was performed in the striatum as detailed in the Supplementary material.

### Isolation of synaptosomes

Striatal synaptosomes were isolated as described previously, with minor modification.^32^ The striatum from all rats was weighed and homogenized with a Dounce glass homogenizer (Thermo Fisher Scientific) in buffer A [HEPES 10 mM, sucrose 0.32 M, MgCl_2_ 1 mM, CaCl_2_ 0.5 mM, EGTA 1 mM and 1 µl/ml protease inhibitor cocktail (pH 7.4), 10% wt/vol], and then using a 26 GA needle. This homogenate was spun down at 1400*g* for 10 min at 4°C, retaining the supernatant, and resuspending the pellet in buffer A and centrifuging it again at 710*g* for 10 min at 4°C. Both supernatants were mixed and spun down at 11 600*g* for 12 min at 4°C. The pellet was again resuspended in buffer A and overlaid on buffer B (HEPES 10 mM, sucrose 1.4 M). After centrifugation at 20 000*g* for 1 h at 4°C, the interphase was collected as the synaptosome sample. The protein content of individual synaptosomes samples was quantified with the PierceTM BCA protein assay kit (Thermo Fisher Scientific).

### Quantitative proteomics by SWATH-MS and the bioinformatics analysis

Bioenergetic and proteomic studies were carried out in isolated striatal synaptosomes of three representative time points post-inoculation based on the immunohistochemical results: the onset of hα-syn overexpression in the SN_pc_ (72 h) and in the striatum (1 week); and the point at which there was a significant reduction in dopaminergic neurons in the SN_pc_ and of dopaminergic terminals in the striatum (4 weeks). Synaptosomal protein extracts derived from striatal samples were subjected to SWATH-MS (sequential window acquisition of all theoretical mass spectra). Individual protein extracts (20 µg) from all experimental groups were subjected to in-gel digestion, peptide purification and reconstitution before the SWATH-MS runs using the TripleTOF 5600+ instrument configured as described previously.^33^ Significantly dysregulated regulatory/metabolic pathways in synaptosomal fractions were identified with Metascape.^34^ The α-syn interactome was obtained from the curated Biological General Repository for Interaction Datasets (BioGRID: https://thebiogrid.org).^35^ The synaptic ontology analysis was performed using the SynGo platform (https://www.syngoportal.org/index.html).^36^ To identify individual proteins from the deregulated protein list with potential links to Parkinson’s disease or neurodegeneration, we performed an extensive literature search (see Supplementary material for further details).

### Seahorse bioenergetics assay

The oxygen consumption rate (OCR) was measured in freshly purified synaptosomes using a Seahorse XF96 extracellular flux analyser (Agilent) as described previously, with some modifications.^37,38^ Synaptosomes were centrifuged at 15 000*g* for 15 min at 4°C and diluted into ionic medium (pH 7.4). Synaptosomes (8 µg per well) were loaded into PEI/Geltrex-coated microplates, centrifuged at 3400*g* for 1 h at 4°C (Beckman Coulter Allegra X-12R centrifuge) and the ionic medium was replaced with incubation medium at 37°C (pH 7.4). The microplate was incubated in a non-CO_2_ incubator (Incudigit) for 10–15 min at 37°C and then loaded into the XF96 extracellular analyser following the manufacturer’s instructions. Mitochondrial respiration, as indicated by the OCR, was monitored simultaneously in real-time throughout the assay by sequential injection of modulators of the mitochondrial electron transport chain: oligomycin (5 µM), carbonyl cyanide-4-(trifluoromethoxy) phenylhydrazone (FCCP, 4 µM) and rotenone/antimycin A (2 µM each). The OCR data represented the mean rates of each measurement cycle, which consisted of a 30 s mixing time, a 30 s waiting time and 3 min of data acquisition. Basal respiration was measured before the first injection (three cycles) and three data-points were obtained following each injection (12 data-points in total). After the assay, the plate was stained with crystal violet (Sigma-Aldrich) for data normalization, and data were analysed using Wave Desktop 2.6 software (Agilent, Santa Clara, CA, USA). Parameters of mitochondrial respiration were obtained from the OCR data (Supplementary material).

### Electron microscopy for ultrastructural morphological analysis

Brains were collected as described in the immunohistological section previously (*n* = 6 EVV, *n* = 4 hα-syn per time point), and serial coronal vibratome sections (50-µm thick, VT1000S, Leica Microsystems) were collected and stored at 4°C in a preserving solution containing 0.03% sodium azide in PBS (pH 7.4) until their use. Striatal coronal sections were processed for pre-embedding immunoperoxidase labelling as described previously, with some modifications.^39^ First, the sections were processed following the TH immunohistochemistry staining protocol described to assess the ultrastructural changes at dopaminergic terminals. The sections were then washed twice in 0.1 M PB and they were post-fixed in 0.5% osmium tetroxide diluted in 0.1 M PB for 15 min. After two washes in 0.1 M PB, the sections were dehydrated in ascending ethanol dilutions for 10 min each, and then in 70% ethanol with 1% uranyl acetate. The sections were subsequently incubated in ethoxypropanol and embedded in epoxy resin (Durcupan™ ACM). Ultra-thin sections from the superficial planes were obtained with an Ultracut S ultramicrotome (Reichert Technologies), and they were finally contrasted with lead citrate and examined on a transmission electron microscope (TEM: Hitachi H-7650 microscope) equipped with an SC1000 Orius CCD camera (Gatan). Digital images were obtained randomly from the dorsal striatum of both hemispheres (∼+0.2 mm from Bregma according to the atlas^31^) at a final magnification of ×15 000 using the Metamorph software (Molecular Devices, San Jose, CA, USA). Images were obtained that avoided blood vessels, occasional grouped areas of myelination and astroglial swelling, to ensure that mainly neurons and synapses were analysed. Image resolution in the *xy* plane was 3.4 nm/pixel and the images were 3284 × 2600 pixels in size. The area per field of view was 104.35 µm^2^.

From each animal, 20 dorsal striatum TEM images were analysed using ImageJ software (NIH) to assess different ultrastructural parameters of dopaminergic fibres and glutamatergic synapses. All the values calculated were averaged across the animals in each group and for each time point (see Supplementary material for further details of the ultrastructural parameters analysed).

### Triple immunofluorescence for TH-LC3B-Lamp1 and TH-Rab5-Rab7 confocal microscopy

Triple immunodetection of either TH, LC3B and Lamp1 or TH, Rab5 and Rab7 was performed as described previously with slight modifications^40^ to assess the nature of the electroclear vesicles within the dopaminergic fibres observed by TEM. Free-floating striatal coronal sections were permeabilized for 1 h at room temperature in blocking solution (4% normal donkey serum or 4% bovine serum albumin in 0.3% PBS-T) and they were then incubated with the corresponding primary antibodies diluted in PBS for 24 h or for 48 h at 4°C when diluted in blocking solution. The following day, the slices were exposed for 1 h and 30 min at room temperature to the corresponding anti-goat, anti-rabbit and anti-mouse secondary antibodies conjugated to Alexa 488, 546 and 647, respectively, protected from the light. The cell nuclei were counterstained with DAPI (1:10 000, Thermo Fisher Scientific), and the sections were then mounted onto glass slides using Vectashield mounting medium (H-1400, Vector Laboratories) and visualized under a Zeiss LSM 800 confocal laser microscope (Carl Zeiss) with a Plan-Apochromat ×63/1.4 numerical aperture oil-immersion objective (Carl Zeiss).

### Statistical analysis

All the statistical analyses were performed using GraphPad Prism 8.0 software (GraphPad Software Inc.). Data distribution for normality was assessed using the Kolmogorov–Smirnov test and variance equality with a Levene’s test. For repeated measures in the stepping test, a Wilcoxon or Friedman test were used, while the open field data were assessed with a two-way ANOVA. For pair-wise comparisons between the means of the hα-syn and EVV groups, an unpaired *t*-test or Mann–Whitney *U*-test was performed when data were parametric or non-parametric, respectively. For multiple comparisons between the means of the hα-syn and EVV groups over time, a one-way ANOVA followed by a Bonferroni’s *post hoc* test or a Kruskal–Wallis and Dunn’s *post hoc* test were performed when data were parametric or non-parametric, respectively. Group data are represented in graphs as the mean ± SEM and significant differences were set at *P* < 0.05.

### Data availability

An extended version of the materials and methods is available in the Supplementary material. Data that support the findings of this study are available from the corresponding author on reasonable request.

## Results

### AAV-mediated overexpression of A53T-hα-syn induced dopaminergic degeneration without motor impairment

**Figure 1:**
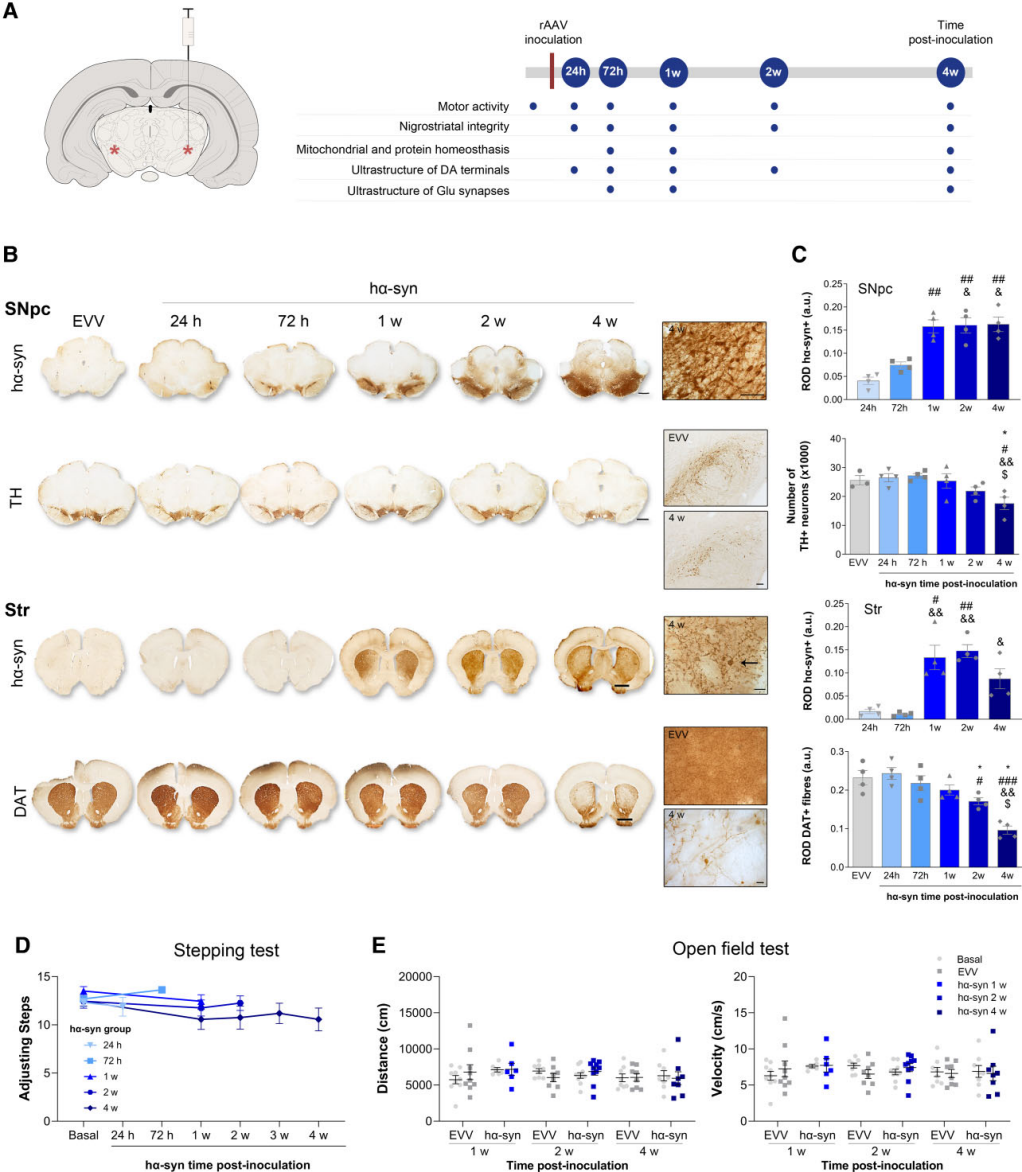
**Evaluation of the animal model**. (**A**) Representation of the viral vector inoculation site (red asterisks, bilaterally in the SN_pc_) and the experimental design indicating the final post-inoculation (p.i.) time points evaluated. (**B**) *Left*: Representative photomicrographs of hα-syn and TH staining in coronal sections of the SN_pc_, and hα-syn and dopamine transporter (DAT) in coronal sections of the striatum from animals that received EVV or hα-syn inoculation assessed at 24 h, 72 h, 1, 2 and 4 weeks (w) post-inoculation Scale bar = 2 mm. *Right*: Representative high magnification photomicrographs showing pathological hα-syn^+^ terminal swellings (arrow) and thickening of fibres at 4 weeks post-inoculation in the striatum. Scale bars = 100 and 10 μm for nigral and striatal sections, respectively. (**C**) The relative optical density (ROD) analysis of hα-syn expression and number of TH neurons in the SN_pc_, and of hα-syn and DAT expression in the striatum: a.u. = arbitrary units. Values are presented as the mean ± SEM (Kruskal–Wallis followed by Dunn’s *post hoc* test): *P < 0.05 versus EVV; ^#^*P* < 0.05, ^##^*P* < 0.01, ^###^*P* < 0.05 versus 24 h post-inoculation; ^&^*P* < 0.05, ^&&^*P* < 0.01 versus 72 h post-inoculation; ^$^*P* < 0.05 versus 1 week post-inoculation *n* = 4 for each group and time point. (**D**) Stepping test for the evaluation of bradykinesia in the hα-syn animals. The values represent the average number of adjusting steps of both forelimbs and they are presented as the mean ± SEM (Friedman or Wilcoxon test within each subgroup, no statistical differences, *n* = 4 for each time point). (**E**) Open field test for the evaluation of the locomotor activity performed before surgery (basal), and at 1, 2 and 4 weeks post-inoculation on EVV and hα-syn inoculated animals. The distance (cm) and velocity (cm/s) values are shown as the mean ± SEM (two-way ANOVA): no statistical differences, *n* = 8 for each group and time point.

We first verified the temporal sequence of hα-syn overexpression and its relationship to motor behaviour and dopaminergic degeneration (Fig. 1A). Significant hα-syn expression in the SN_pc_ and striatum occurred from 1 week post-inoculation onwards (*P* < 0.01: Fig. 1B). The presence of α-syn^+^ Lewy body-like structures in the striatum was evident at 4 weeks post-inoculation (Fig. 1Band 1C) concomitantly with the significant bilateral loss of dopaminergic neurons in the SN_pc_ (31% cell loss versus EVV, *P* < 0.05: Fig. 1C). A significant loss of TH^+^ and dopamine transporter positive fibres in the dorsal striatum respect to the EEV group was observed from 2 weeks post-inoculation onwards (25% *P* < 0.05 and 22% *P* < 0.05 at 2 weeks post-inoculation, respectively; 48% *P* < 0.01 and 55% *P* < 0.05 at 4 weeks post-inoculation, respectively: Fig. 1B and C and Supplementary Fig. 1), but no significant changes were observed in the nucleus accumbens (NAc) (Supplementary Fig. 1). This dopaminergic degeneration was not associated with impairment in motor activity as no significant changes were observed in either the stepping or the open field test (Fig. 1D and E).

#### Overexpression of hα-syn in the SN_pc_ causes early proteostasis changes in striatal synapses

**Figure 2:**
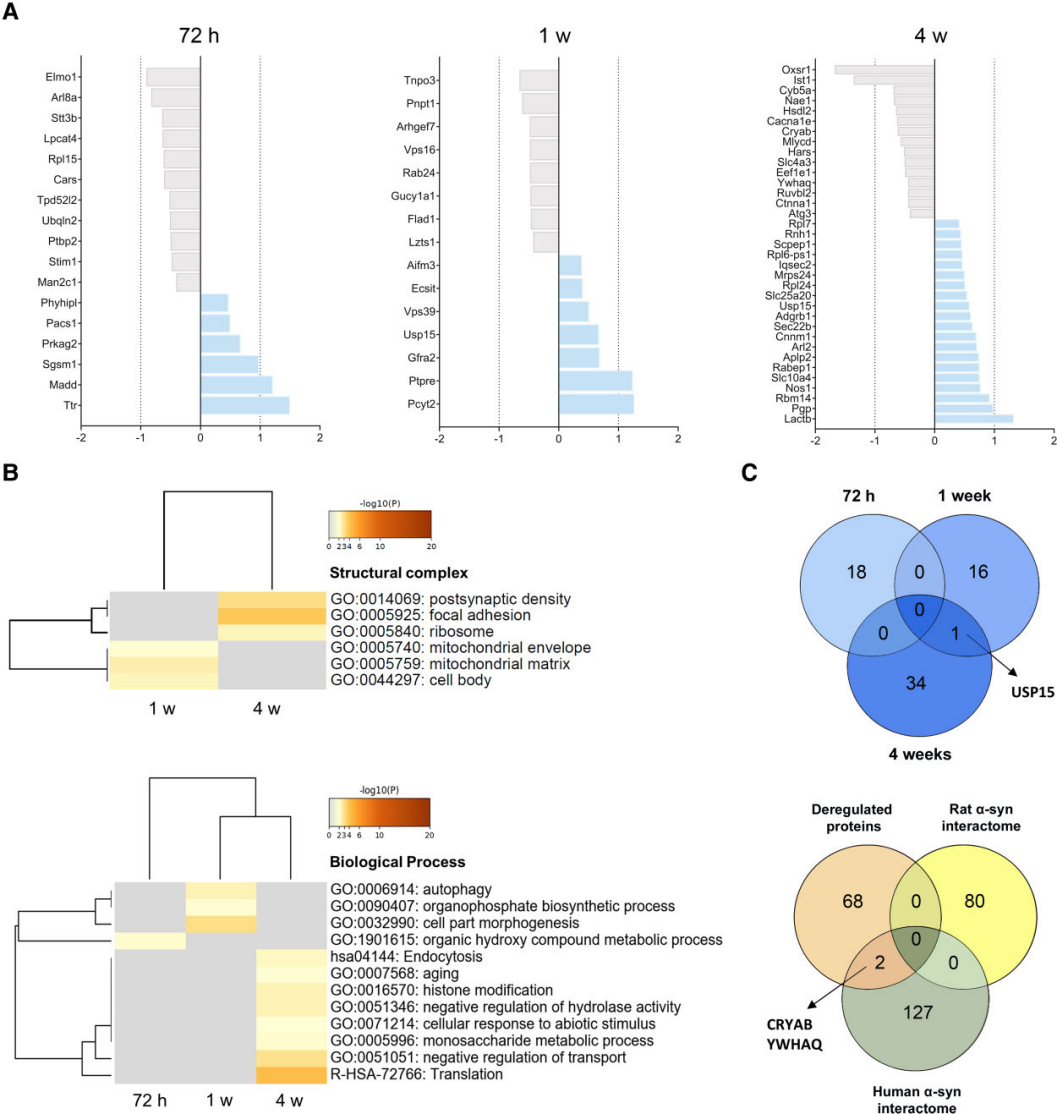
**Protein homeostasis of striatal synaptosomes**. (**A**) Significantly deregulated proteins in hα-syn inoculated striatal synaptosomes at 72 h, 1 week and 4 weeks post-inoculation The grey and blue colours indicate downregulated and upregulated proteins, respectively. (**B**) Heat map showing the top enrichment terms (GO) across the post-inoculation time points using a colour scale to represent statistical significance. The grey colour indicates a lack of significance. *Top*: Enriched structural complexes across the 1 and 4 weeks post-inoculation time points of the hα-syn rats. No enriched structural complex was observed at 72 h post-inoculation. *Bottom*: Enriched biological pathway and gene ontology clusters in the hα-syn inoculated animals at different post-inoculation time points (**C**) *Top*: Cluster overlap of deregulated proteins at striatal synaptosomes at the different post-inoculation time points (72 h, 1 week and 4 weeks) after hα-syn overexpression. The Usp15 protein overlapped across the 1 and 4 weeks post-inoculation *Bottom*: Cluster overlap between deregulated proteins in the hα-syn animals and the α-syn interactomes from both rat and human. Two of the deregulated proteins, CRYAB and YWHAQ, overlapped with the hα-syn interactome (*n* = 5 per group and time point).

A total of 2298 individual proteins were identified by SWATH-MS proteomics, of which 68 proteins were significantly deregulated by hα-syn when all the different time points were considered (Fig. 2A and Supplementary Table 1). Of these proteins, 15 were mapped to SynGO annotated genes database.^36^ This deep synaptic gene ontology analysis revealed that protein changes occurred at the presynaptic and postsynaptic levels, involving proteins partially participating in protein translation or in synaptic signalling and organization Supplementary Table 2).

The earliest change observed occurred at 72 h post-inoculation, where 18 differential expressed proteins were obtained (Fig. 2C). Some of these proteins were associated with organic hydroxyl compound metabolism (TTR, DPM1, STT3B, PRKAG2), including proteins involved in glycosylation and metabolic stress. At the same time, no association was obtained through the synaptic structural complex (Fig. 2B). At 1 week post-inoculation, 16 significantly deregulated proteins were observed (Fig. 2C), mainly linked to mitochondria and involved in cell part morphogenesis (GFRA2, ARHGEF7, LZTS1, PNPT1 and PTPRE), autophagy (LZTS1, VPS39, VPS16 and RAB24) and metabolic processes like organophosphate biosynthetic processes (GUCY1A1, PCYT2 and FLAD1: Fig. 2B). At 4 weeks post-inoculation, there were 35 deregulated proteins mainly related to ribosomes, the postsynaptic density and focal adhesions, consistent with an involvement in biological processes like translation (HARS1, RPL7, RPL24, EEF1E1, MRPS24, RABEP1, YWHAQ, IQSEC2, ATG3, MLYCD and RUVBLL2), negative regulation of transport (CRYAB, NOS1, OXSR1, YWHAQ, ATG3, CACNA1E, CYB5A and SLC10A4), endocytosis (RABEP1, IST1 and IQSEC2), ageing, histone modification, negative regulation of hydrolase activity, cellular responses to abiotic stimulus and monosaccharide metabolic process (Fig. 2B and C). It should be noted that proteins involved in the postsynaptic density were only observed at this last time point.

Based on the clustering analysis, only one upregulated protein overlapped across 1 and 4 weeks post-inoculation [ubiquitin carboxyl-terminal hydrolase 15 (USP15); Fig. 2C]. This protein plays a critical role in the removal of ubiquitin from substrates and it has been proposed to oppose Parkin-mediated mitophagy.^41^ A comparison of all the significantly deregulated proteins following hα-syn overexpression with the rat and hα-syn interactomes indicated no overlap with the rat α-syn interactome, while the CRYAB and YWHAQ proteins downregulated at 4 weeks post-inoculation overlapped with the hα-syn interactome (Fig. 2C). CRYAB acts as a molecular chaperone that primarily binds to misfolded proteins to prevent aggregation, while YWAHQ is an adapter protein implicated in the regulation of a wide range of signalling pathways. Both these proteins have been proposed to be interactors of hα-syn experimentally.^42,43^

These findings indicate that synaptic homeostasis is altered very soon after hα-syn overexpression, initially by proteins involved in bioenergetic regulation, yet closely followed by proteins participating in the clearance of proteins and in translation. Proteins implicated in the postsynaptic density are only altered late following the previous modifications.

### Mitochondrial ultrastructural abnormalities in striatal dopaminergic terminals occur at late stage

**Figure 3:**
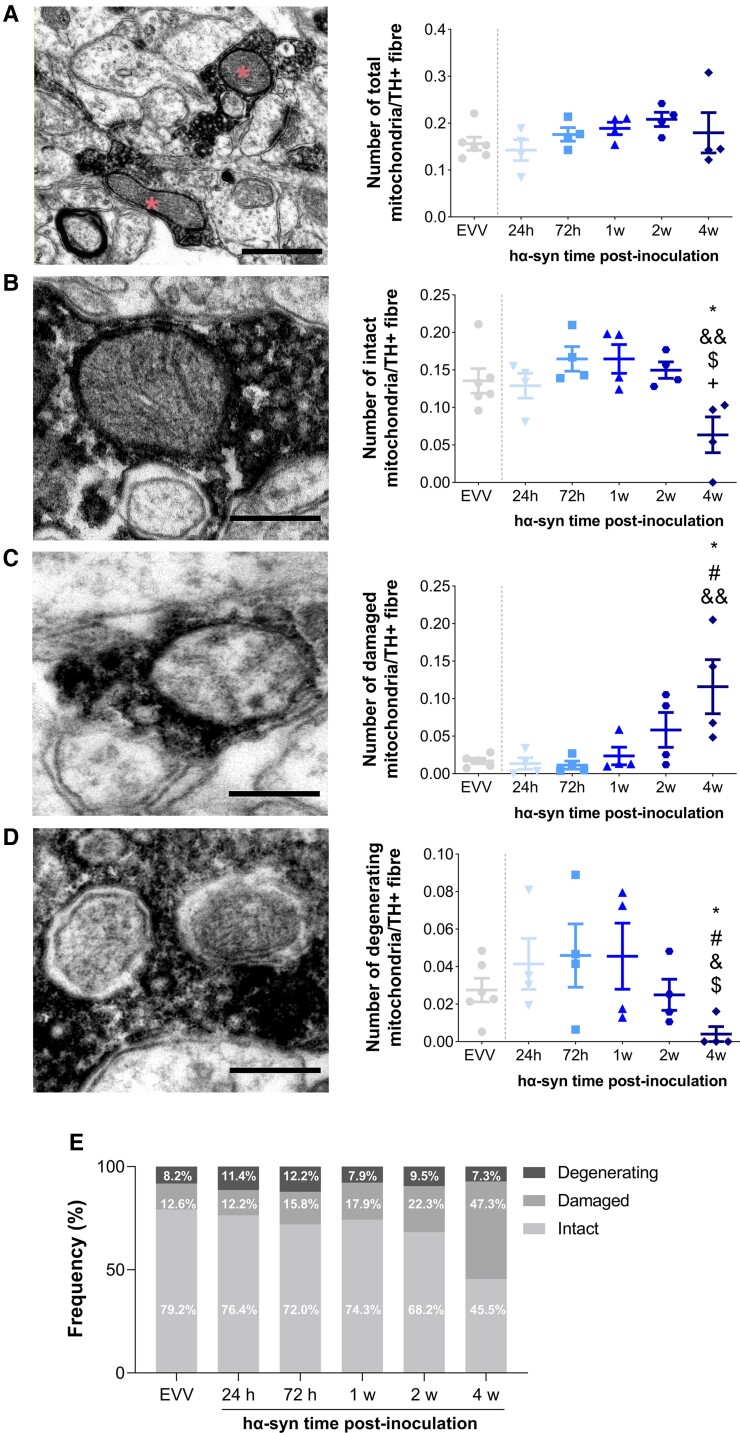
**Ultrastructure of the mitochondria of TH+ dopaminergic fibres**. (**A**) *Left*: Electron microscopy photomicrographs of mitochondria (red asterisks) inside TH^+^ fibres (black staining) in the dorsal striatum. Scale bar = 0.5 µm. *Right*: The number of mitochondria per TH^+^ fibre 24 h, 72 h, 1, 2 and 4 weeks after EVV or hα-syn inoculation. *Left*: Representative electron microscopy photomicrograph of intact (**B**), damaged (**C**) and degenerating (**D**) mitochondria inside a TH^+^ fibre (black staining). Scale bar = 200 nm. Right, the number of intact (**B**), damaged (**C**) and degenerating (**D**) mitochondria per TH^+^ fibre 24 h, 72 h, 1, 2 or 4 weeks after EVV or hα-syn inoculation. All values represent the mean ± SEM (Kruskal–Wallis followed by Dunn’s *post hoc* test): *P < 0.05 versus EVV; ^#^*P* < 0.05 versus 24 h; ^&^*P* < 0.05 versus 72 h; ^&&^*P* < 0.01 versus 72 h; ^$^*P* < 0.05 versus 1 week post-inoculation; ^+^*P* < 0.05 versus 2 weeks post-inoculation. (**E**) Proportion of intact, damaged and degenerating mitochondria inside TH^+^ fibres after EVV or hα-syn inoculation (*n* = 6 EVV, *n* = 4 hα-syn for each time point).

Subsequently, we studied mitochondrial ultrastructural abnormalities in striatal dopaminergic axon terminals. At 1 week post-inoculation there was no change in the overall number of mitochondria in the striatal TH^+^ fibres, in the number of mitochondrial subtypes (intact, damaged or degenerating mitochondria) (Fig. 3A and E) and in the major descriptors of total mitochondrial shape (Supplementary Fig. 2).

At 4 weeks post-inoculation, although the overall number of mitochondria in striatal TH^+^ fibres was unchanged, the density of intact mitochondria was lower (*P* < 0.05 versus EVV at 1 and 2 weeks post-inoculation, *P* < 0.01 versus 72 h post-inoculation: Fig. 3B), the density of damaged mitochondria was increased (*P* < 0.05 versus EVV and 24 h post-inoculation; *P* < 0.01 versus 72 h post-inoculation: Fig. 3C) and the density of degenerating mitochondria was lower (*P* < 0.05 versus EVV at 24 h, 72 h and 1 week post-inoculation: Fig. 3D). Interestingly, we also observed an increase in the length-to-width ratio (*P* < 0.01 versus 24 h post-inoculation and *P* < 0.05 versus 2 weeks post-inoculation) and a decrease in the roundness (*P* = 0.070 versus EVV, *P* < 0.01 versus 24 h post-inoculation, and *P* < 0.05 versus 2 weeks post-inoculation) of intact mitochondria at 4 weeks post-inoculation (Supplementary Fig. 2).

### Dopaminergic fibres exhibit autophagic/endocytic disturbances before the ultrastructural changes in the late stage

**Figure 4:**
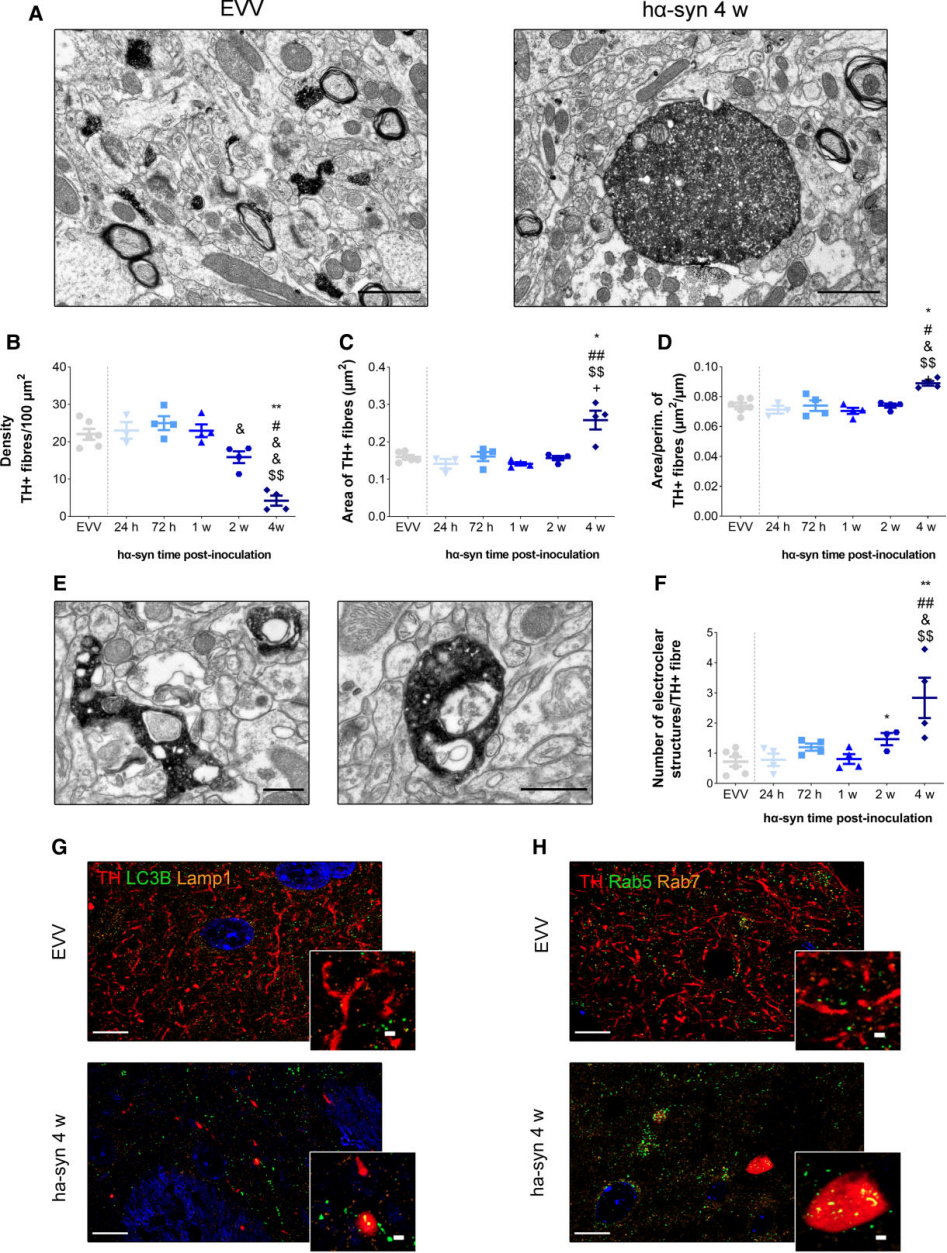
**Ultrastructure of TH^+^ dopaminergic fibres and electroclear structures within TH^+^ dopaminergic fibres**. (**A**) Representative electron microscopy photomicrographs of stained TH^+^ fibres (black) in the dorsal striatum 4 w after EVV or hα-syn inoculation. Scale bars = 1 µm. Density (**B**), area (µm), (**C**) and mean area/perimeter ratio (µm^2^/µm) (**D**) of TH^+^ fibres 24 h, 72 h, 1, 2 and 4 weeks after EVV and hα-syn inoculation. All values represent the mean ± SEM (Kruskal–Wallis followed by Dunn’s *post hoc* test): *P < 0.05 and **P < 0.01 versus EVV; ^#^*P* < 0.05 and ^##^*P* < 0.01 versus 24 h; ^&^*P* < 0.05 and ^&&^*P* < 0.01 versus 72 h; ^$$^*P* < 0.01 versus 1 week post-inoculation; ^+^*P* < 0.05 versus 2 weeks post-inoculation (*n* = 6 EVV, *n* = 4 hα-syn for each time point). (**E**) Representative electron microscopy photomicrographs of electroclear structures found in TH^+^ fibres. Scale bars = 200 nm. (**F**) Number of electroclear structures per TH^+^ fibre 24 h, 72 h, 1, 2 and 4 weeks after EVV and hα-syn inoculation (Kruskal–Wallis followed by Dunn’s *post hoc* test): *P < 0.05 and **P < 0.01 versus EVV; ^##^*P* < 0.01 versus 24 h; ^&^*P* < 0.05 versus 72 h; ^$$^*P* < 0.01 versus 1 week post-inoculation (*n* = 6 EVV, *n* = 4 hα-syn for each time point). (**G** and **H**) Expression of autophagic and endocytic proteins within TH^+^ dopaminergic fibres. Representative triple immunofluorescence photomicrographs for TH (red), LC3B (green) and Lamp1 (orange), (**G**) and TH (red), Rab5 (Green), Rab7 (orange) (**H**) 4 weeks after EVV and hα-syn inoculation. Scale bars = 10 µm and 1 µm for the higher magnification photomicrographs (DAPI, blue).

Ultrastructural examination in the hα-syn group confirmed the reduction of striatal dopaminergic innervation and evidenced axon pathology in the remaining dopaminergic fibres (Fig. 4A). Thus, we observed a significant and progressive reduction of TH^+^ fibres at 2 weeks (*P* < 0.05 versus 72 h post-inoculation) and 4 weeks post-inoculation (*P* < 0.01 versus EVV, 72 h and 1 week post-inoculation; *P* < 0.05 versus 24 h post-inoculation: Fig. 4B). The remaining TH^+^ fibres had a significant increase in size (*P* < 0.05 versus EVV and 2 weeks post-inoculation; *P* < 0.01 versus 72 h and 1 week post-inoculation: Fig. 4C) and an increase in their area/perimeter ratio at 4 weeks post-inoculation, (*P* < 0.05 versus EVV, 24 h, 72 h and 2 weeks post-inoculation; *P* < 0.01 versus 1 week post-inoculation: Fig. 4D and Supplementary Table 4).

There was also a significant increase in the number of electroclear structures at 2 weeks post-inoculation (*P* < 0.05 versus EVV) and 4 weeks post-inoculation (*P* < 0.01 versus EVV, 24 h and 1 week post-inoculation; *P* < 0.05 versus 72 h post-inoculation: Fig. 4F). These structures were characterized by a discernible electron-lucent lumen that could contain different cell structures (Fig. 4E) but did not show other morphological changes (Supplementary Table 5). To characterize the nature of these electroclear structures inside the TH^+^ fibres, we studied the expression of key endocytic (Rab5 and Rab7) and autophagic (LC3B and Lamp1) proteins at 4 weeks post-inoculation There was an accumulation of LC3 and Rab5 but no Rab7 and Lamp1 expression in the swollen TH^+^ fibres relative to the EVV controls (Fig. 4G and H).

### Postsynaptic target redistribution and plastic changes in excitatory synapses in the striatum

**Figure 5:**
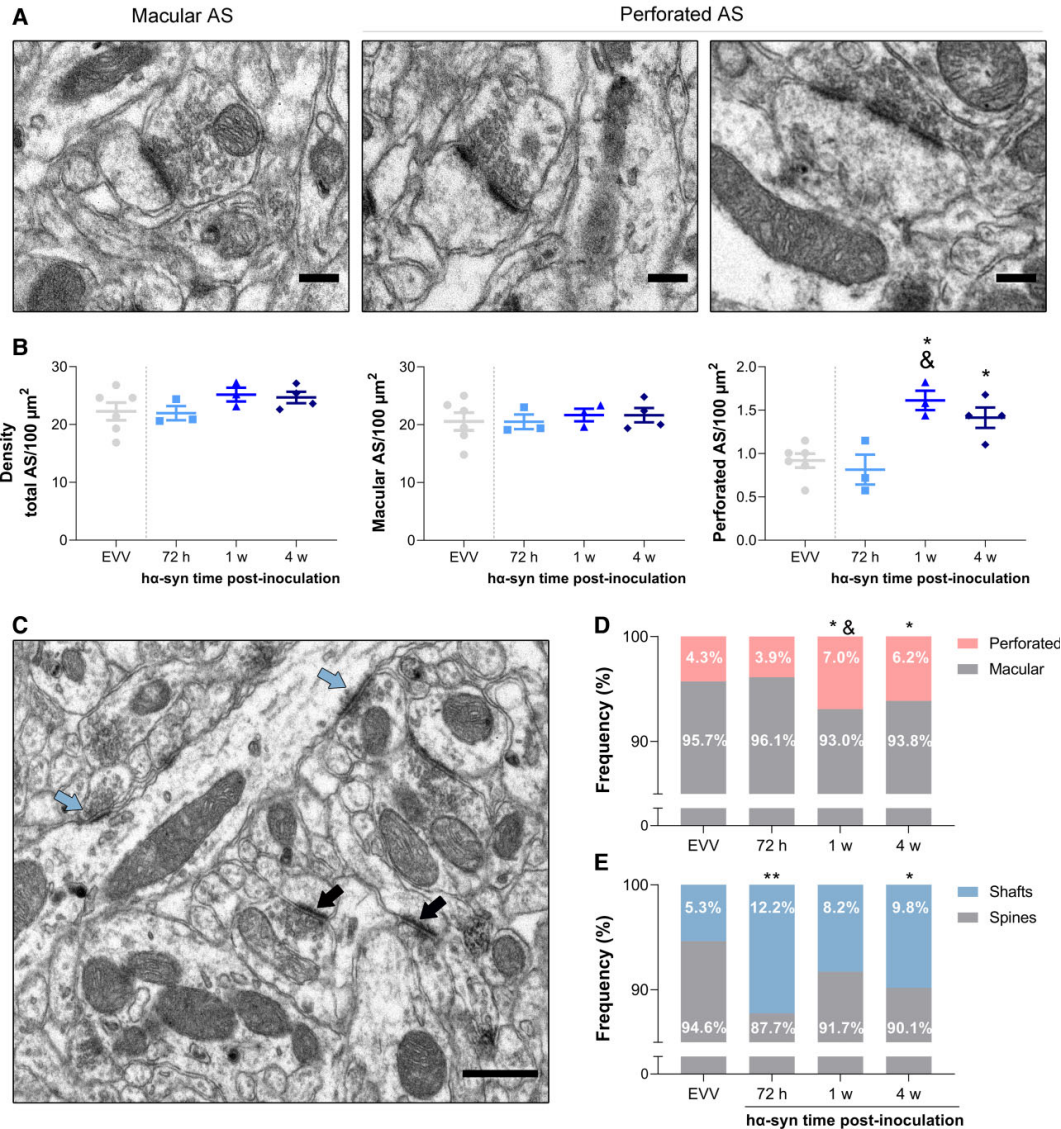
**Density of ASs in the dorsal striatum**. (**A**) Electron microscopy photomicrographs from the dorsal striatum of a macular and perforated ASs with single and dual perforation. Scale bars = 200 nm. (**B**) Density of total, macular and perforated ASs per 100 µm^2^ after EVV and hα-syn inoculation. All values are presented as the mean ± SEM (Kruskal–Wallis followed by Dunn’s *post hoc* test): *P < 0.05 versus EVV; ^&^*P* < 0.05 versus 72 h post-inoculation. (**C**) Electron microscopy photomicrograph showing ASs on dendritic spines (black arrows) and dendritic shafts (blue arrows). Scale bar = 0.5 µm. (**D**) Proportion of perforated and macular ASs (% total ASs). (**E**) Proportion of ASs on dendritic spines and dendritic shafts (Kruskal–Wallis followed by Dunn’s *post hoc* test for each type of target): *P < 0.05 and **P < 0.01 versus EVV (*n* = 6, EVV; *n* = 3, 72 h; *n* = 3, 1 week; *n* = 4, 4 weeks).

The density and morphology of the presynaptic terminal, postsynaptic density (PSD) and postsynaptic targets of glutamatergic ASs within the dorsal striatum were also studied. No significant differences in the density of ASs were found (Fig. 5B). When classifying ASs into macular or perforated synapses (Fig. 5A), this last type being considered structural intermediates in synaptic plasticity, the number of perforated synapses was increased at 1 week post-inoculation (*P* < 0.05 versus EVV and 72 h post-inoculation) and 4 weeks post-inoculation (*P* < 0.05 versus EVV: Fig. 5B and D). To corroborate possible mechanisms of homeostatic plasticity associated to these ultrastructural changes, we analysed the expression of AMPA receptors (GluA1 and GluA2/3) on PSD fractions from striatal isolated synaptosomes by western blotting. No differences in GluR1 and GluR2/3 levels were observed (Supplementary Fig. 4). No morphological differences were observed at the presynaptic level of these ASs (Supplementary Table 6). Conversely, there was a slight increment in the number of mitochondria within glutamatergic presynaptic terminals at 4 weeks post-inoculation (*P* < 0.05 versus EVV), but no alterations in mitochondrial morphology were evident (Supplementary Fig. 3).

We did not find any alterations to the length of the PSD of either total ASs, or macular and perforated PSDs (Supplementary Table 6). We further analysed the two main postsynaptic targets: dendritic spines (forming axospinous synapses) and dendritic shafts (forming axodendritic synapses: Fig. 5C). There was a reduction in the proportion of axospinous synapses and an increase in the proportion of axodendritic synapses at 72 h (*P* < 0.01 versus EVV) and at 4 weeks post-inoculation (*P* < 0.05 versus EVV: Fig. 5E).

### Mitochondrial respiration at striatal synapses is early altered and becomes compromised at later stages

**Figure 6:**
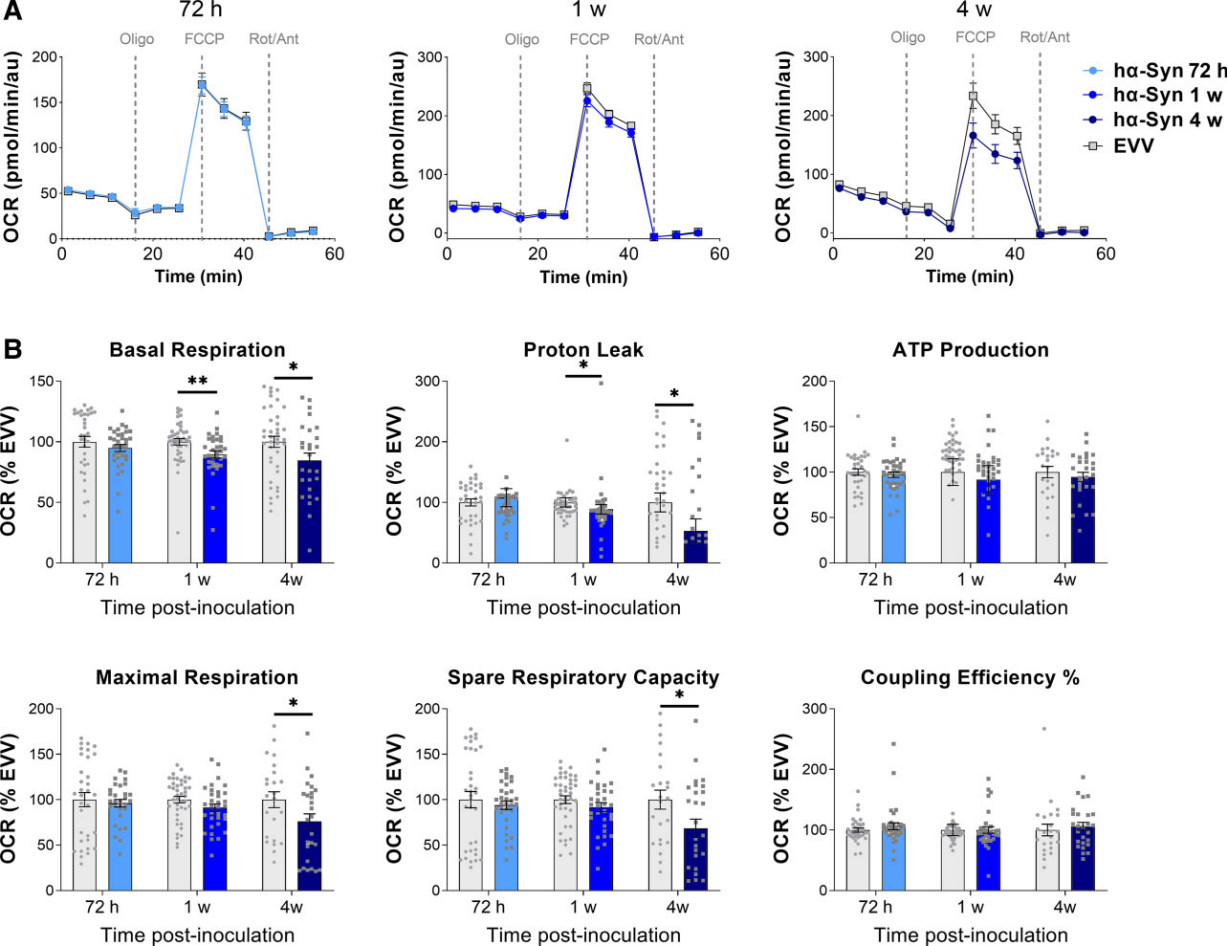
**Mitochondrial bioenergetics of striatal synaptosomes**. (**A**) Normalized OCRs (pmol/min/au) in synaptosomes from EVV and hα-syn inoculated animals at 72 h, 1 week and 4 weeks post-inoculation after sequential injections of oligomycin, FCCP and rotenone/antimycin A. (**B**) Normalized bioenergetic parameters derived from results in **A**, representing basal respiration, proton leak, ATP production, maximal respiration, spare respiratory capacity and coupling efficiency (%). Three independent experiments, one for each time point (72 h, 1 week and 4 w post-inoculation): *n* = 3 animals per group and time point, and *n* = 8–12 wells per animal. Data are presented as the mean ± SEM in arbitrary units (au): *P < 0.05; **P < 0.01 versus relative to the EVV group (Mann–Whitney *U*-test).

A significant decrease was detected in the OCR bioenergetic profile of isolated striatal synaptosomes of the hα-syn rats at 1 and 4 weeks post-inoculation compared their corresponding EVV group (Fig. 6A). Reduced basal respiration and proton leak (Fig. 6B) was observed at 1 week post-inoculation (*P* < 0.01 and *P* < 0.05, respectively) and at 4 weeks post-inoculation (*P* < 0.05: Fig. 6B). In addition, at 4 weeks post-inoculation there was a significant decrease in the maximal respiration (*P* < 0.05) and spare respiratory capacity (*P* < 0.05: Fig. 6B), while no such differences were observed in the rest of the bioenergetic parameters analysed (ATP production and percentage coupling efficiency, Fig. 6B).

To determine whether these alterations to mitochondrial respiration might be due to the presence of fewer mitochondria, we examined the expression of several fundamental mitochondrial proteins based on the quantitative proteomic data. No quantitative changes in the protein subunits of the translocase of the mitochondrial outer membrane (TOM), NADH dehydrogenase complex (complex I), cytochrome *c* oxidase (complex IV) or ATP synthase (complex V) were detected at any time point. Moreover, there were no alterations to the expression of proteins related to mitochondrial dynamics (fission and fusion), such as mitofusin 2 (MFN2) and optic strophy 1 (OPA1), or in the recruitment of the mitochondrial fission protein dynamin 1 (DNM1L) at any of the time points (Supplementary Table 3).

## Discussion

We have studied for the first time the earliest changes in the striatal dopaminergic synapses induced by hα-syn overexpression showing how functional alterations are primary events preceding structural changes and loss of dopaminergic neurons at a premotor stage. We describe that mitochondrial dysfunction and energetic failure is the earliest event followed by autophagic/endocytic flux dysfunction and accumulation of dysfunctional organelles preceding synaptic structural changes and cell death. We also describe the proteins involved in such changes that could be potential therapeutic targets to counterpart synaptic malfunction and prevent degeneration. Changes in striatal glutamatergic synapse, accounting for structural plasticity and potentially helping the maintenance of motor function, are also observed adding value to this model of premotor parkinsonism and to our findings.

The earliest alteration to the striatal synapses (72 h post-inoculation), involves the deregulation of proteins affecting the metabolism. Synaptic activity is tightly coupled to metabolic energy, requiring tight control of the energy balance for correct neuronal activity.^4–6,47^ The upregulation of PRKAG2, a sensor protein of the cell energy status, reflects a state of metabolic stress and it suggests that hα-syn overexpression rapidly triggers an energy deficit that accentuates thereafter.^48^ This condition of stress is also supported by the fact that transthyretin (TTR), the most strongly upregulated protein at this time, is a marker of oxidative stress.^49,50^ Therefore, these early proteostatic alterations in synaptic metabolism could initiate a cascade of pathological events leading to the bioenergetic dysfunctions that starts at 1 week post-inoculation At this time point, a deregulation of proteins related to the mitochondrial compartment (ACAD8, PNPT1, AIFM3 and ECSIT) commences being the upregulation of ECSIT particularly notable as it responds to signs of oxidative stress or mitochondrial damage, triggering the activation of protective molecular events like mitophagy.^51^ However, the mitochondrial clearance system might be disrupted as USP15 is a deubiquitinating enzyme that catalyses the removal of ubiquitin from substrates opposing Parkin-mediated mitophagy.^42^ The upregulation of USP15 at 1 and 4 weeks post-inoculation suggests a possible inhibition of mitophagy, which would coincide with the onset of mitochondrial respiratory defects (1 week post-inoculation) and that might cause the ensuing mitochondrial ultrastructural changes and energetic compromise (4 weeks post-inoculation). Remarkably, this occurs without any reduction in the fundamental mitochondrial proteins often used to study mitochondrial content, such as TOM and cytochrome *c*, and the number of mitochondria at dopaminergic presynaptic terminals, as well as the mitochondrial subtypes, do not appear to suffer ultrastructural alterations at this time point (1 week post-inoculation). Thus, our findings confirm that these early bioenergetic alterations were due to a uniquely functional deficit in respiration and not to changes in mitochondrial content or structure. It is important to notice that dopaminergic neurons are especially vulnerable to degeneration in Parkinson’s disease. Although the ultimate reason for this is unknown, these neurons have a high-energy demand that has been considered relevant.^5,6^ Interestingly, we observe that although hα-syn is overexpressed in the whole striatum, dopaminergic degeneration mainly occurs in the dorsal region, which is where these findings are observed. This indicates that in this model the dopaminergic neurons of the ventro-lateral tier of the SN_pc_ (projecting mostly to the dorsal striatum) are especially vulnerable as these occur in patients with Parkinson’s disease, supporting our results. This could be due to their extensive axonal arbour and consequent high bioenergetics demand making them more vulnerable to stressors such as α-syn accumulation.^5,6,50,52^ Thus, we demonstrate that even modest alterations in synaptic mitochondrial activity in these dopaminergic neurons conveys dramatic changes and therefore, altered energy supply/demand could initiate the cascade of events leading to the neurodegeneration associated with Parkinson’s disease.^5,6^

Modulation of some of these proteins related to the mitochondrial compartment aiming to restore bioenergetics to preserve synaptic function could be a potential therapeutic strategy. In this regard, recent studies stimulating the clearance of damaged mitochondria in patients with Parkinson’s disease^53,54^ and other neurodegenerative diseases,^55^ as well as in animal models of parkinsonism or fibroblasts from idiopathic Parkinson’s disease patients have shown promising results.^54,56^ Additionally, other small-molecules enhancing mitophagy have been described as potential therapeutic targets in parkinsonian models.^42,54^ Thus, in the same line, we postulate that inhibition of USP15 activity with small molecule inhibitors could selectively re-engage homeostatic redox responses as has been previously suggested with other similar deubiquitinating enzymes.^57^ As far as mitochondrial deficits have been described in other neurodegenerative diseases, our findings could be relevant to test in other animal models, which could potentially provide early therapeutic targets for such disorders.^55^

At 4 weeks post-inoculation, a reduction in spare respiratory capacity was observed, which has been considered a major factor that defines neuron survival.^37,58^ Thus, this indicates that synaptic mitochondria are already functionally impaired, compromising the synaptic function and structure accounting for dopaminergic loss. Indeed, following mitochondrial dysfunction there is a loss of dopaminergic axon terminals from the second week of hα-syn overexpression onwards being significant at 4 weeks post-inoculation when ultrastructural changes associated with the degeneration of axons are also present. This indicates that the remaining dopaminergic fibres acquire a pathological conformation with an observable swollen and bulging morphology, in keeping with earlier studies showing swollen TH^+^ fibres in the striatal terminals of post-mortem brains of Parkinson’s disease patients ^59,60^ and in animal models of Parkinson’s disease.^61,62^ Moreover, although the total mitochondrial population is maintained, there is an increase in damaged mitochondria at 4 weeks post-inoculation Interestingly, the remaining intact mitochondria have morphological features related to enhanced activity (increment in length-to-width ratio and a decrease in the roundness),^63^ which, although speculative, might indicate a functional compensation through morphological changes to potentially support continued neural transmission. Furthermore, several proteins linked to ribosomes and protein translation were seen to be deregulated, indicating that the imbalance in the regulation of local protein translation might contribute to synaptic dysfunction at this time point.^64^

Correct synaptic autophagy is also critical to maintain dopaminergic synapses.^65–67^ We found that from the second week onwards there is a significant increase in the number of electroclear vesicles within dopaminergic fibres, which co-localize with LC3^+^ and Rab5^+^ at 4 weeks post-inoculation, markers of autophagy and endocytic vesicles, respectively. This is also witnessed by the deregulation of proteins involved in autophagic flux from 1 week post-inoculation and the deregulation of proteins related to endocytosis and transport at 4 weeks post-inoculation. Of these proteins, the upregulation of VPS39 suggests an impairment in the endosomal maturation cycle.^68^ Additionally, it has been described that Ist1 and ATG3 inhibition represses autophagic flux^69^ and RABEP1^70^ and SECC22B^71^ overexpression triggers the accumulation of large endocytic vesicles.^70^ Accordingly, a sequence of early proteostatic changes in the autophagic/endocytic system could result in impaired maturation of autophagy intermediates, which may then lead to an accumulation of endosomes and autophagy cargo within the dopaminergic terminals, contributing to neurodegeneration.^72^ We now demonstrate that mitochondrial and autophagic dysfunction are at play in the earliest phase of dopaminergic degeneration, preceding motor function defects.

By comparing the hα-syn interactome with our deregulated proteome we found CRYAB and YWHAQ were significantly downregulated at 4 weeks post-inoculation. Interestingly, this finding coincides with the onset of dopaminergic cell body degeneration. CRYAB is a chaperone that act as a potent inhibitor of α-syn amyloid fibril formation, preventing aggregation and its ensuing neurotoxicity.^73,74^ YWHAQ or the 14-3-3θ protein also has a chaperone function and it reduces α-syn toxicity and propagation.^75,76^ Thus, we speculate that the decrease in CRYAB and 14-3-3θ are directly related to hα-syn accumulation and that it may be a critical mechanism by which α-syn propagation and toxicity arise in Parkinson’s disease.

Another interesting aspect of our study is the observation that glutamatergic synapses in the dorsal striatum undergo complex ultrastructural remodelling already observed in neurotoxic models of parkinsonism and that has been claimed as a sign of homeostatic plasticity.^18,77–79^ Similar to our findings, ultrastructural remodelling features have been observed in advanced stages of dopaminergic depletion^18,23,79–81^ and in post-mortem brains of Parkinson’s disease.^82^ However, there are no previous studies in the early stage of dopaminergic degeneration. The early decrease in the proportion of axospinous synapses at 72 h, which is also later observed at 4 weeks post-inoculation, along with the proteostatic alterations to the PSD after hα-syn overexpression, might be contributing to maintaining stable levels of excitability, reflecting a process analogous to what has been defined as synaptic homeostatic scaling, whereby the strength of a neuron’s postsynaptic inputs are adjusted to compensate for extended changes in overall activity and stabilizing regional synaptic weighting.^83^ This is also consistent with the increase in the density of perforated ASs observed from 1 week post-inoculation onwards, without alterations to the overall or macular AS density. Perforated synapses have been described as structural intermediates in synaptic turnover^84,85^ and their larger PSD perimeter surface leads to more efficient neurotransmission than in macular synapses.^86,87^ In addition, the increase in the number of mitochondria within glutamatergic terminals provides further evidence for the possible enhanced glutamatergic activity that could potentially sustain the mechanisms of plasticity observed as suggested in other studies.^88^ Last, although the increase of GluR1 and GluR2/3 is related to homeostatic plasticity, our study did not show such a rise in PSD fractions. These results are in keeping with previous studies in the striatum of parkinsonian rats where no changes in GluR were found.^89–92^ Actually, it is thought that they only occur after long-lasting dopaminergic depletion explaining our findings.

We also observe that proteins involved in spine formation such as Elmo1^93^ are downregulated at 72 h post-inoculation, and cell part morphological proteins that are down- and upregulated have been found at 1 week post-inoculation suggestive of structural remodelling at the synaptic level as has been described in analogous studies where α-syn overexpression leads to abnormalities in spine dynamics and impairments on dendritic spine stability and plasticity.^94,95^ Likewise, the decrease in axospinous glutamatergic synapses of spiny projection neurons in Parkinson’s disease patients^96,97^ and animal models of advanced parkinsonism^23,81,98,99^ have been associated with spine pruning. Future studies addressing the striatal spine plasticity and its functional significance in the early stages of nigrostriatal degeneration should help better understand these plastic mechanisms.

These adaptive changes in excitatory synapses might occur in an attempt to maintain the synaptic strength and the normal striatal activity despite deficiencies progressively produced by dopaminergic modulation. Although we have not studied dopaminergic transmission in our model and being one of the limitations of this study, impaired dopaminergic neurotransmission has been described as an early synaptic event that precedes neuronal degeneration in the same animal model.^27^

It is important to note that our functional and proteomic studies are performed on isolated striatal nerve terminals that are a mixture of both dopaminergic and glutamatergic synapses as no immunopurification was performed based on synaptic surface markers. In addition, long flow cytometry sorting times would be needed to accumulate sufficient material. However, ultrastructural studies were performed separately on dopaminergic and glutamatergic terminals, and data obtained by both approaches were consistent, although it would be important to perform future studies on sorted synaptic populations.

Overall, we describe a progressive striatal synaptopathy in the premotor stages of dopaminergic degeneration that opens new avenues for targeting key synapse proteins to develop disease-modifying therapies aimed at regaining synaptic function in this prodromal phase. Our data pave the way for future development of synapse-targeted therapies to be used in patients at risk of Parkinson’s disease (i.e. genetic forms, patients with REM sleep behaviour disorders or hyposmia) and hopefully in the future in participants with reliably early biomarkers.

## Supplementary Material

awac087_Supplementary_Data

## Abbreviations

α-syn =: α-synuclein
AS: asymmetric synapse
EVV: empty vector
OCR: oxygen consumption rate
ROD: relative optical density
SN_pc_: substantia nigra pars compacta
