# Supplementary material for: Striatal synaptic bioenergetic and autophagic decline in premotor experimental parkinsonism
Source: Brain. 2022 Mar 4;145(6):2092–107. doi: 10.1093/brain/awac087 (PMC9460676; doi:10.1093/brain/awac087)
Supplement: awac087_Supplementary_Data [file awac087_supplementary_data.pdf]

Supplementary Figures

Fig. S1

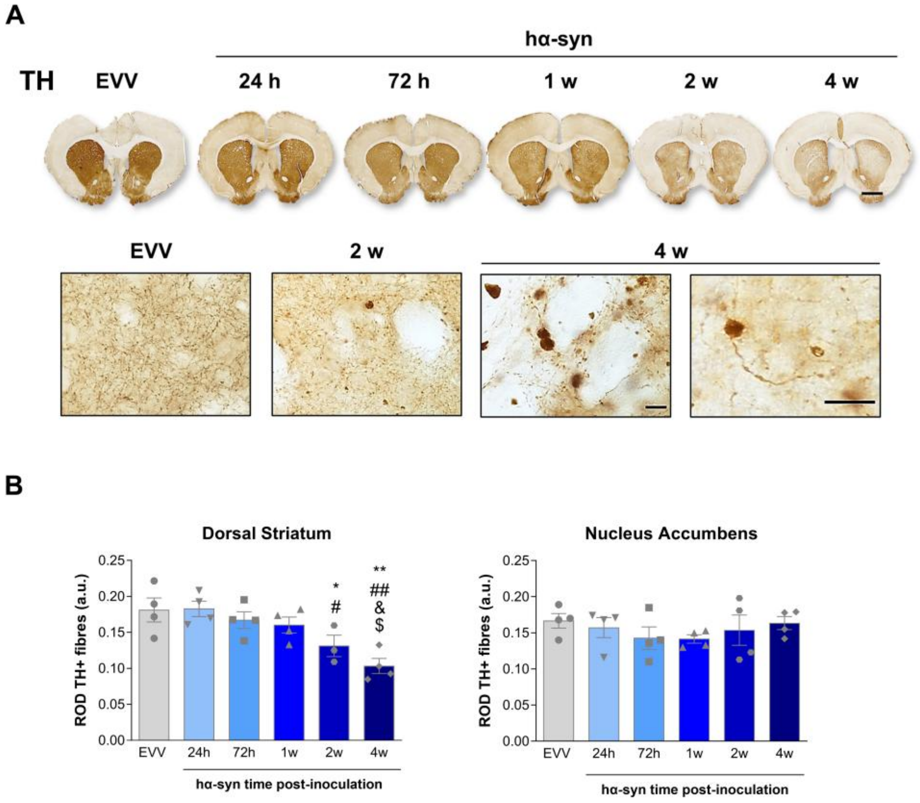

Supplementary Figure 1. TH expression in the striatum. (**A**) Top, Representative striatal photomicrographs for TH staining in coronal sections from EVV and hα-syn groups at 24 h, 72 h, 1, 2 and 4 weeks p.i., scale bar 2 mm. Bottom, Higher magnification photomicrographs of TH staining. Scale bars, 10 µm. (**B**) Relative optical density (ROD) values of TH expression in the EVV and hα-syn groups. All values are presented as mean ± SEM. Kruskal-Wallis followed by Dunn´s post-hoc test: * $P < 0.05$ and ** $P < 0.01$ vs. EVV group; # $P < 0.05$ and ##· $P < 0.01$ vs. 24 h; & $P < 0.05$ vs. 72 h; $ $p < 0.05$ vs. 1 w p.i. $n = 4$ for each group and time point.

Fig. S2

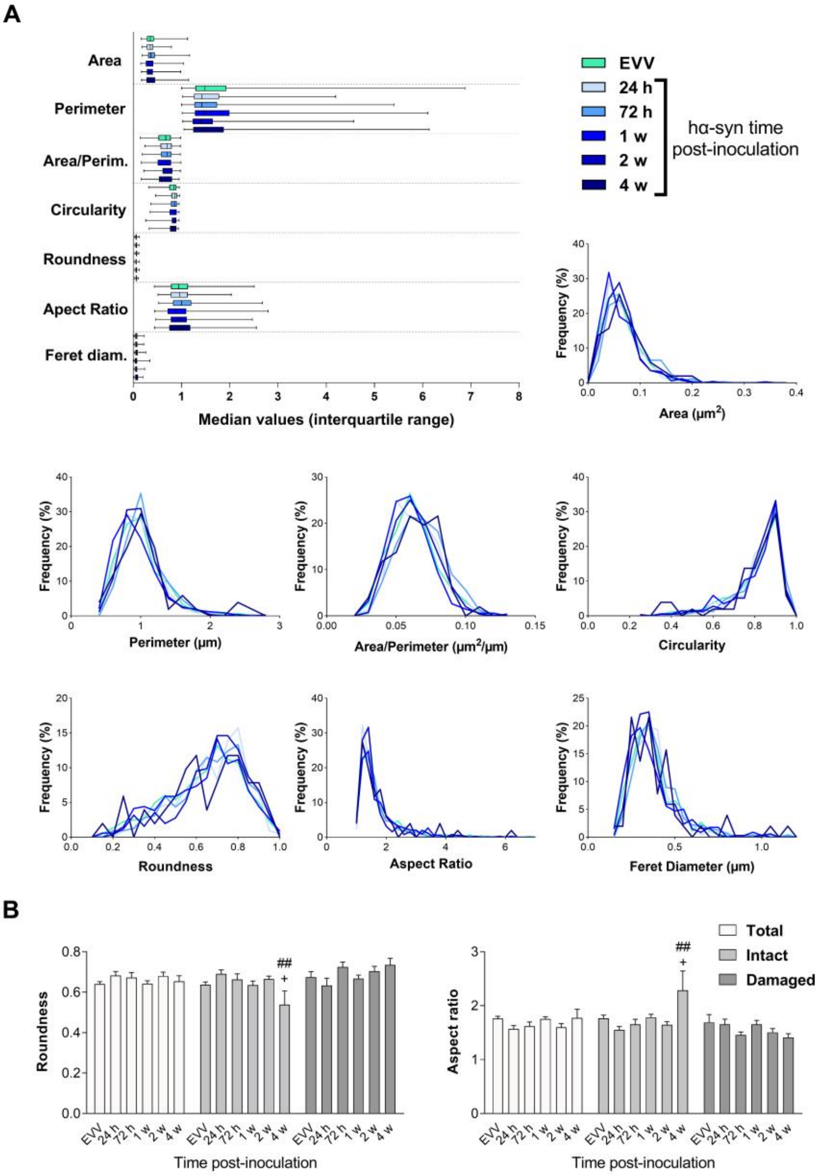

Supplementary Figure 2. Morphological analysis of mitochondria found inside TH+ fibres. (**A**) Top, Box plot graph representing total mitochondrial morphological parameters from EVV and hα-syn group time points. Bars represent the range of values that contain 50% of all mitochondria (interquartile range) for each parameter. Bottom, Frequency distribution histograms (% total mitochondria) for each morphological parameter of mitochondria: area, perimeter, area/perimeter ratio, circularity, roundness, aspect ratio, and Feret diameter. No statistical differences in the means (Kruskal-Wallis test). *n*= 447 (EVV), *n*= 217 (24 h), *n* = 323 (72 h), *n* = 340 (1 w), *n* = 253 (2 w), *n*= 51 (4 w) mitochondria. (**B**) Analysis of average roundness and aspect ratio of the total, intact and damaged mitochondria inside TH+ fibres from EVV and hα-syn groups. Values are presented as mean ± SEM. Kruskal-Wallis followed by Dunn´s post-hoc test for each type of mitochondria: ## $P < 0.01$ vs. 24 h; + $P < 0.05$ vs. 2 w p.i. $n = 6$ (EVV), $n = 4$ (hα-syn group for each time point).

Fig. S3

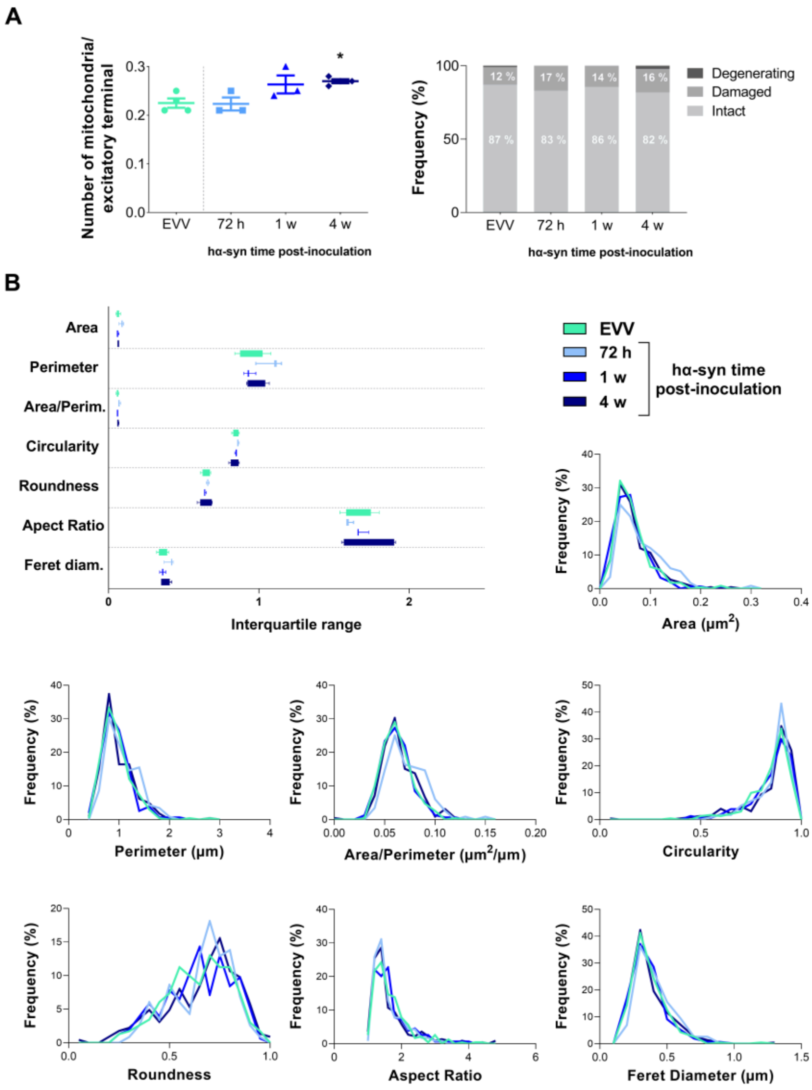

Supplementary Figure 3. Analysis of density and morphology of mitochondria in excitatory presynaptic terminals. (**A**) Left, Number of mitochondria per excitatory presynaptic terminal in EVV and hα-syn groups. All values are presented as mean ± SEM. Kruskal-Wallis followed by Dunn´s post hoc test: * $P < 0.05$ vs. EVV group. n = 6 (EVV); n = 3 (72 h); n = 3 (1 w); n = 4 (4 w) animals. Right, Proportion of intact, damaged and degenerating mitochondria (% total mitochondria) in EVV and hα-syn groups. (**B**) Top, Box plot graph representing mitochondrial morphological parameters from EVV and hα-syn groups. Bars represent the range of values that contain 50% of all mitochondria (interquartile range) for each parameter. Bottom, Frequency distribution histograms (% total mitochondria) for each morphological parameter: area, perimeter, area/perimeter ratio, circularity, roundness, aspect ratio and Feret diameter. No statistical differences in the means (Kruskal-Wallis test). n = 231 (EVV), n = 116 (72 h); n = 154 (1 w); n = 225 (4 w) mitochondria.

Fig. S4

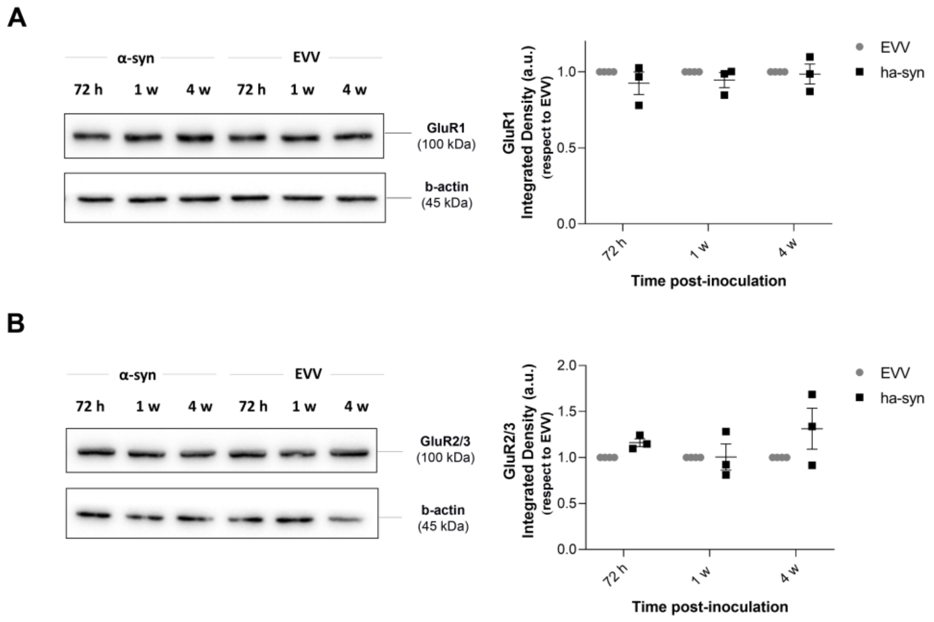

Supplementary Figure 4. Expression of GluR1 and GluR2/3 receptors in the PSD fraction of striatal isolated synaptosomes. Representative western blot showing receptors expression and integrated density values of (**A**) GluR1 and (**B**) GluR2/3 at 72h, 1 week, and 4 weeks in the EVV and hα-syn groups. β-actin was used as the loading control. All values are presented as mean ± SEM. No statistical differences in the means (Kruskal-Wallis test followed by Dunn´s post hoc test.). *n* = 3 (72 h); *n* = 3 (1 w); *n* = 3 (4 w) in EVV and hα-syn groups.

Supplementary table 1: Significantly deregulated proteins in striatal synaptosomes at 72 h, 1 w and 4 w after hα-syn inoculation

Description	Gene Name	Uniprot	Peptide Count	Ratio	p Value
**72 h**					
Downregulated proteins					
Dolichol-phosphate mannosyltransferase subunit 1	DPM1	D4A8N1	2	0.5153	0.0226
Engulfment and cell motility 1	ELMO1	G8CYZ7	2	0.5335	0.0090
ADP-ribosylation factor-like GTPase 8A	ARL8A	D3ZPP2	2	0.5645	0.0254
RCG25591, isoform CRA_a	STT3B	B2RYD7	2	0.6409	0.0005
Lysophosphatidylcholine acyltransferase 4	LPCAT4	D3ZR52	5	0.6434	0.0038
60S ribosomal protein L15	RPL15	P61314	4	0.6520	0.0094
Cysteinyl-tRNA synthetase	CARS	G3V9K0	4	0.6554	0.0143
Tumor protein D54	TPD52L2	Q6PCT3	4	0.6965	0.0285
Ubiquilin 2	UBQLN2	D4AA63	4	0.7012	0.0012
Polypyrimidine tract-binding protein 2	PTBP2	Q66H20	2	0.7063	0.0029
Stromal interaction molecule 1	STIM1	A0A0G2K5C8	3	0.7163	0.0171
Alpha-mannosidase 2C1	MAN2C1	Q5M9I2	7	0.7535	0.0396
Up-regulated proteins					
Phytanoyl-CoA hydroxylase-interacting protein-like	PHYHIPL	Q6AYN4	6	1.3875	0.0356
Phosphofurin acidic cluster sorting protein 1	PACS1	F1LPG3	10	1.4131	0.0315
Protein kinase AMP-activated non-catalytic subunit gamma 2	PRKAG2	A0A140UHX4	2	1.5921	0.0100
Small G protein-signalling modulator 1	SGSM1	D3ZAS2	2	1.9722	0.0140
MAP kinase-activating death domain protein	MADD	A0A0G2KA27	7	2.3184	0.0078
Transthyretin	TTR	P02767	3	2.8239	0.0194

Description	Gene Name	Uniprot	Peptide Count	Ratio	p Value
**1 week**					
Downregulated proteins					
Acyl-CoA dehydrogenase family, member 8	ACAD8	M0RDK9	2	0.5381	0.0473
Transportin 3	TNPO3	D4AAM0	2	0.6327	0.0171
Polyribonucleotide nucleotidyltransferase 1	PNPT1	G3V6G7	2	0.6530	0.0440
Rho guanine nucleotide exchange factor 7	ARHGEF7	A0A0G2QC21	4	0.7137	0.0436
Vacuolar protein sorting-associated protein 16 homolog	VPS16	Q642A9	3	0.7149	0.0335
RAB24, member RAS oncogene family	RAB24	A0A096MKB0	3	0.7161	0.0308
Guanylate cyclase soluble subunit alpha-1	GUCY1A1	P19686	7	0.7191	0.0414
FAD synthase	FLAD1	D4A4P4	3	0.7229	0.0028
Leucine zipper putative tumor suppressor 1	LZTS1	Q8CFC9	2	0.7432	0.0288
Up-regulated proteins					
Apoptosis-inducing factor, mitochondria-associated 3	AIFM3	D3ZF03	6	1.3116	0.0232
Evolutionarily conserved signaling intermediate in Toll pathway	ECSIT	Q5XIC2	2	1.3211	0.0425
VPS39 subunit of HOPS complex	VPS39	E9PT04	4	1.4222	0.0145
Ubiquitin carboxyl-terminal hydrolase	USP15	A0A0A0MY07	2	1.5970	0.0219
GDNF family receptor alpha-2	GFRA2	O35977	2	1.6091	0.0336
Receptor-type tyrosine-protein phosphatase epsilon	PTPRE	B2GV87	2	2.3642	0.0021
Ethanolamine-phosphate cytidylyltransferase	PCYT2	O88637	2	2.4023	0.0308
**4 weeks**					
Downregulated proteins					
Oxidative stress-responsive kinase 1	OXSR1	D3ZUC9	2	0.3131	0.0298
IST1 homolog	IST1	Q568Z6	2	0.3902	0.0162
Cytochrome b5	CYB5A	P00173	3	0.6194	0.0215
NEDD8-activating enzyme E1 regulatory subunit	NAE1	F1M7W7	4	0.6229	0.0497
Hydroxysteroid dehydrogenase-like protein 2	HSDL2	Q4V8F9	6	0.6376	0.0013
Voltage-dependent R-type calcium channel subunit alpha	CACNA1E	F1LMS1	2	0.6460	0.0474

Description	Gene Name	Uniprot	Peptide Count	Ratio	p Value
Alpha-crystallin B chain	CRYAB	P23928	2	0.6496	0.0231
Malonyl-CoA decarboxylase, mitochondrial	MLYCD	Q920F5	4	0.6732	0.0242
Dead end homolog 1	HARS	Q4QQV4	3	0.6996	0.0244
Anion exchange protein	SLC4A3	G3V8P8	6	0.7041	0.0061
Eukaryotic translation elongation factor 1 epsilon 1	EEF1E1	B2RYN3	2	0.7085	0.0225
14-3-3 protein theta	YWHAQ	P68255	11	0.7325	0.0070
RuvB-like helicase	RUVBL2	G3V8T5	2	0.7333	0.0413
Catenin alpha 1 isoform CRA_b	CTNNA1	Q5U302	10	0.7341	0.0180
Ubiquitin-like-conjugating enzyme ATG3	ATG3	Q6AZ50	3	0.7485	0.0378
Up-regulated proteins					
60S ribosomal protein L7	RPL7	B0K031	7	1.3351	0.0283
Ribonuclease inhibitor	RNH1	E2RUH2	10	1.3571	0.0213
Retinoid-inducible serine carboxypeptidase	SCPEP1	Q920A6	2	1.3696	0.0220
60S ribosomal protein L6	RPL6-PS1	F1LQS3	5	1.3790	0.0407
IQ motif and Sec7 domain ArfGEF 2	IQSEC2	A0A0G2JZX5	9	1.3809	0.0324
Mitochondrial ribosomal protein S24	MRPS24	A9UMV2	2	1.4151	0.0353
60S ribosomal protein L24	RPL24	A0A0H2UH99	4	1.4250	0.0411
Mitochondrial carnitine/acylcarnitine carrier protein	SLC25A20	P97521	6	1.4584	0.0061
Ubiquitin carboxyl-terminal hydrolase	USP15	A0A0A0MY07	2	1.4977	0.0391
Adhesion G protein-coupled receptor B1	ADGRB1	C0HL12	4	1.5214	0.0253
Vesicle-trafficking protein SEC22b	SEC22B	Q4KM74	7	1.5500	0.0016
Cyclin M1	CNNM1	D4A1C0	2	1.6207	0.0114
ADP-ribosylation factor-like protein 2	ARL2	O08697	2	1.6340	0.0271
Amyloid-like protein 2	APLP2	M0RDX2	2	1.6732	0.0280
Rab GTPase-binding effector protein 1	RABEP1	G3V9J7	3	1.6860	0.0199
Sodium/bile acid cotransporter 4	SLC10A4	F1LQG5	3	1,6865	0.0407
Nitric oxide synthase	NOS1	F1LQL1	2	1.7050	0.0091
RCG48334, isoform CRA_e (RNA-binding motif protein 14)	RBM14	M0R9Q1	2	1.8935	0.0394
Glycerol-3-phosphate phosphatase	PGP	D3ZDK7	2	1.9772	0.0398
β-Lactamase	LACTB	D3ZFJ6	6	2.5059	0.0370

Supplementary table 2: Synaptic ontologies of the hα-syn deregulated proteome at 72 h, 1 w and 4 w p.i.

Ontology term	Protein count	Corresponding time point of proteins	p-value	q-value
**Cellular component**				
synapse	13	2 (72 h), 2 (1 w), 9 (4 w)	3.77e-5	2.26e-4
presynapse	8	2 (1 w), 6 (4 w)	3.32e-4	9.95e-4
postsynapse	8	1 (72 h), 1 (1 w), 6 (4 w)	1.29e-3	1.93e-3
presynaptic ribosome	3	1 (72 h), 2 (4 w)	1.01e-3	1.93e-3
postsynaptic ribosome	3	1 (72 h), 2 (4 w)	2.30e-3	2.76e-3
postsynaptic density	4	1 (1 w), 3 (4 w)	0.0120	0.0120
**Biological Process**				
process in the synapse	12	2 (72 h), 3 (1 w), 7 (4 w)	2.41e-5	1.20e-4
protein translation at presynapse	3	1 (72 h), 2 (4 w)	9.62e-4	2.07e-3
protein translation at postsynapse	3	1 (72 h), 2 (4 w)	1.24e-3	2.07e-3
synaptic signaling	3	1, (1 w), 2 (4 w)	0.0233	0.0291
synapse organization	3	2 (1 w), 1 (4 w)	0.0829	0.0829

The protein count column shows the number of proteins annotated in SynGO for each term.

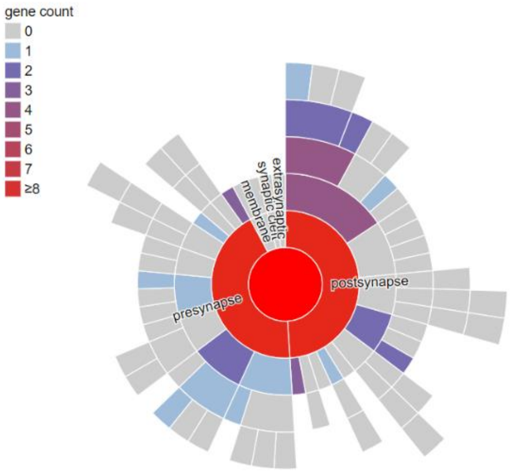

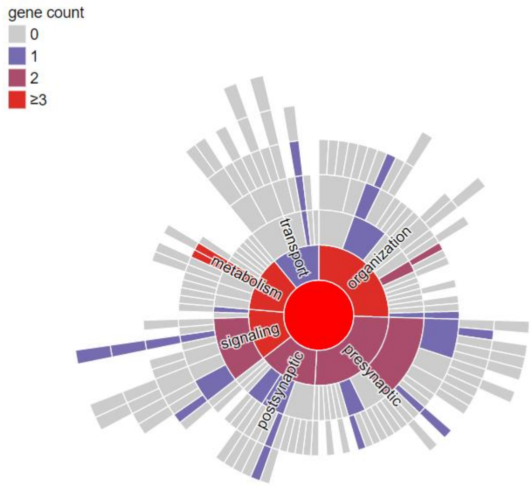

Supplementary table 3: Description of the mitochondrial proteins detected by SWATH-MS proteomics in striatal synaptosomes 72 h, 1 w and 4 w after hα-syn and EVV inoculation

	Gene	Uniprot	Peptide count	72 h		1 w		4 w	
				Ratio	p Value	Ratio	p Value	Ratio	p Value
**Mitochondrial import receptors**	Tomm70	Q75Q39	17	0.997	0.967	0.926	0.611	1.028	0.644
	Tomm34	Q3KRD5	8	1.019	0.737	1.135	0.571	1.007	0.907
	Tomm40	G3V8F5	6	0.906	0.308	0.927	0.757	1.066	0.367
	Tomm22	Q75Q41	2	0.925	0.510	0.959	0.867	1.137	0.260
**NADH dehydrogenase (Complex I)**	Ndufs1	Q66HF1	19	1.024	0.683	0.945	0.444	0.998	0.962
	Ndufv1	Q5XIH3	15	0.970	0.561	0.971	0.815	1.044	0.388
	Ndufa10	Q561S0	14	0.997	0.945	0.948	0.590	0.926	0.148
	Ndufs2	Q641Y2	15	1.003	0.936	0.995	0.962	1.020	0.647
	Ndufa9	Q5BK63	14	1.006	0.915	0.960	0.631	1.035	0.572
	Ndufs3	D3ZG43	10	1.029	0.685	0.984	0.788	0.979	0.632
	Ndufb10	D4A0T0	10	1.001	0.982	0.995	0.958	1.037	0.553
	Ndufv2	P19234	8	1.016	0.775	0.975	0.782	1.072	0.409
	Ndufa13	D3ZE15	5	0.938	0.327	0.938	0.438	1.080	0.225
	Ndufa5	Q63362	5	1.171	0.204	1.030	0.826	0.997	0.967
	Ndufa12	F1LXA0	5	1.059	0.122	0.971	0.858	1.004	0.951
	Ndufs7	Q5RJN0	3	1.020	0.699	1.016	0.904	1.035	0.456
	Ndufb7	D3ZLT1	4	0.998	0.971	0.964	0.652	1.101	0.144
	Ndufs4	Q5XIF3	4	1.092	0.161	1.030	0.827	1.053	0.434
	Ndufaf7	Q5XI79	3	1.100	0.555	1.140	0.604	0.952	0.616
	Ndufv3	G3V644	3	1.539	0.185	0.915	0.929	1.117	0.612
	Ndufaf1	F1LWG4	4	0.760	0.065	0.940	0.733	0.979	0.857
	Ndufb9	B2RYW3	4	0.973	0.663	1.001	0.994	1.029	0.642
	Ndufb5	D4A565	6	1.026	0.609	0.993	0.939	1.022	0.622
	Ndufa7	A9UMV9	5	1.049	0.693	0.948	0.792	1.012	0.842
	Ndufs8	B0BNE6	5	0.951	0.436	0.991	0.911	1.025	0.717
	Ndufa6	D4A3V2	2	0.981	0.849	0.966	0.881	1.048	0.514
	Ndufaf2	A0A0G2JZF6	3	1.122	0.418	0.748	0.417	1.071	0.531
	Ndufs5	B5DEL8	3	1.091	0.514	0.990	0.923	1.098	0.146
	Ndufa8	A0A0G2JVL6	5	0.981	0.649	1.010	0.920	0.983	0.748
	Ndufa2	D3ZS58	2	0.923	0.494	0.972	0.902	1.005	0.942
	Ndufaf4	Q9NQR8	2	1.003	0.975	0.851	0.787	1.014	0.946
	Ndufc2	Q5PQZ9	3	1.007	0.812	0.999	0.994	1.062	0.437
	Ndufa4	B2RZD6	2	1.133	0.096	0.912	0.344	0.898	0.088
	Ndufb4	F1LPG5	3	1.041	0.376	1.028	0.860	1.020	0.727
	Ndufb8	B2RYS8	4	0.967	0.490	1.000	1.000	1.075	0.148
	Ndufaf3	O08776	3	1.098	0.433	0.850	0.687	1.171	0.256
	Ndufab1	D3ZF13	2	0.831	0.284	0.780	0.515	0.951	0.633
	Ndufa11	Q80W89	3	0.972	0.700	0.981	0.816	1.042	0.615
	Ndufb6	D3ZZ21	2	0.907	0.261	0.948	0.698	1.125	0.071
	Ndufb11	D4A7L4	2	1.128	0.285	0.977	0.944	1.006	0.934
	Ndufb3	D4A4P3	2	0.830	0.468	1.030	0.924	1.186	0.406
	Ndufaf6	A0A0H2UI06	2	1.064	0.880	1.000	1.000	1.008	0.987

**Cytochrome c oxidase (Complex IV)**	Cox4i1	P10888	7	1.059	0.435	0.968	0.763	0.948	0.299
	Cox6b1	D3ZD09	5	1.061	0.371	0.982	0.861	1.008	0.848
	Cox5b	P12075	6	1.016	0.893	1.038	0.827		0.265
	Cox5a	P11240	4	1.041	0.662	0.891	0.438	1.014	0.836
	Cox7a2l	D3ZYX8	3	0.976	0.693	1.042	0.651	1.018	0.799
**ATP synthase (Complex V)**	Atp5f1a	P15999	18	1.056	0.485	0.975	0.888	0.911	0.032
	Atp5f1b	G3V6D3	18	1.019	0.790	0.991	0.966	0.948	0.360
	Atp5pb	P19511	10	1.080	0.419	0.944	0.572	1.010	0.891
	Atp5pd	P31399	9	1.071	0.268	0.982	0.891	1.006	0.898
	Atp5po	Q06647	7	1.017	0.613	0.951	0.651	1.024	0.658
	Atp5f1c	Q6PCU0	6	1.037	0.506	0.958	0.645	1.005	0.908
	Atpaf2	D3ZTW7	2	0.845	0.336	1.305	0.667	1.605	0.173
	Atp5mg	Q6PDU7	2	0.877	0.312	0.934	0.796	1.089	0.302
	Atp5me	P29419	4	1.019	0.720	0.938	0.446	1.045	0.219
	Atp5f1d	G3V7Y3	2	1.225	0.296	0.786	0.697	0.885	0.506
	Atp5mf	D3ZAF6	2	0.985	0.783	0.944	0.622	0.949	0.163
	Atp8	P11608	2	1.150	0.294	0.930	0.726	0.985	0.873
**Citrate synthase**	Cs	G3V936	12	1.067	0.334	0.995	0.980	0.924	0.037
**Mitochondrial fusion and fission proteins**	Mitofusin-2	Q6IRL2	13	0.959	0.384	0.991	0.970	1.078	0.339
	Opa1	Q2TA68	20	1.030	0.525	0.954	0.641	0.983	0.708
	Dnm1l	O35303	20	1.063	0.278	0.958	0.775	0.935	0.099

Supplementary table 4: Detailed density and morphological parameters of TH$^+$ fibres after EVV and hα-syn inoculation .

	TH$^+$ fibres					
	**EVV**	**24 h**	**72 h**	**1 w**	**2 w**	**4 w**
**Density TH$^+$ fibres per 100 μm^2**	22.024 ± 1.431	23.030 ± 2.203	24.987 ± 1.856	22.950 ± 1.688	15.871 ± 1.593	4.228 ± 1.342 **
**Morphology**						
No of fibres analysed	2758	1442	2084	1916	1412	353
**Area (μm^2)**						
Mean ± SEM	0.159 ± 0.005	0.141 ± 0.012	0.160 ± 0.012	0.141 ± 0.004	0.156 ± 0.005	0.258 ± 0.025 *
Range	0.144 - 0.176	0.166 - 0.155	0.126 - 0.181	0.132 - 1.152	0.140 - 0.166	0.187 - 0.305
**Perimeter (μm)**						
Mean ± SEM	1.858 ± 0.055	1.664 ± 0.114	1.826 ± 0.082	1.712 ± 0.044	1.797 ± 0.107	2.137 ± 0.164
Range	1.689 - 2.040	1.493 - 1.815	1.01 - 1.978	1.611 -1.822	1.506 - 2.022	1.853 - 2.552
**Area/perim. (μm^2/μm)**						
Mean ± SEM	0.073 ± 0.002	0.071 ± 0.002	0.074 ± 0.003	0.070 ± 0.002	0.073 ± 0.001	0.089 ± 0.001 *
Range	0.066 - 0.079	0.067 - 0.076	0.067 - 0.082	0.065 - 0.075	0.070 - 0.076	0.086 - 0.093

* $p < 0.05$ and ** $p < 0.01$ vs. EVV group.

Supplementary table 5: Detailed density and morphological parameters of electroclear structures found inside TH$^+$ fibres after EVV and hα-syn inoculation.

	Electroclear structures inside TH$^+$ fibres					
	**EVV**	**24 h**	**72 h**	**1 w**	**2 w**	**4 w**
**Struc./TH$^+$ f**	0.724 ± 0.065	0.785 ± 0.101	1.203 ± 0.059	0.823 ± 0.072	1.233 ± 0.138 *	2.837 ± 0.334 **
**Area (µm^2)**						
Mean ± SEM	0.013 ± 0.002	0.011 ± 0.001	0.011 ± 0.001	0.010 ± 0.002	0.012 ± 0.001	0.019 ± 0.011
Range	0.007 - 0.024	0.007 - 0.016	0.007 - 0.013	0.007 - 0.018	0.011 - 0.015	0.005 - 0.052
**Perimeter (µm)**						
Mean ± SEM	0.389 ± 0.037	0.352 ± 0.030	0.325 ± 0.010	0.352 ± 0.033	0.367 ± 0.022	0.390 ± 0.095
Range	0.280 - 0.530	0.280 - 0.431	0.299 - 0.350	0.300 - 0.444	0.335 - 0.432	0.256 - 0.673
**Area/perim. (µm^2/µm)**						
Mean ± SEM	0.022 ± 0.002	0.021 ± 0.001	0.019 ± 0.001	0.020 ± 0.002	0.019 ± 0.001	0.018 ± 0.003
Range	0.016 - 0.029	0.016 - 0.024	0.016 - 0.022	0.016 - 0.026	0.018 - 0.022	0.014 - 0.028

* $p < 0.05$ and ** $p < 0.01$ vs. EVV group.

Supplementary table 6: Number of asymmetric synapses (ASs) and detailed morphological data regarding the area (μm^2), perimeter (μm) and area/perimeter ratio (μm^2/μm) of excitatory presynaptic terminals and postsynaptic density length after EVV and hα-syn inoculation.

	Asymmetric Synapses			
	**EVV**	**72 h**	**1 w**	**4 w**
**Density ASs/100 μm^2**	22.27	21.97	25.17	24.68
**Number ASs**	2687	1314	1458	1924
% Macular	95.72 % (2572)	96.12 % (1263)	93.07 % (1357)	93.87 % (1806)
% Perforated	4.28 % (115)	3.88 % (51)	6.93 % (101)*	6.13 % (118)*
**Presynaptic terminal**				
**Area (μm^2)**				
Mean ± SEM	0.238 ± 0.019	0.268 ± 0.014	0.252 ± 0.005	0.246 ± 0.010
Range	0.204-0.311	0.243-0.294	0.247-0.263	0.218-0.266
**Perimeter (μm)**				
Mean ± SEM	2.360 ± 0.075	2.384 ± 0.051	2.380 ± 0.028	2.341 ± 0.060
Range	2.147-2.600	2.311-2.483	2.344-2.437	2.183-2.460
**Area/perimeter (μm^2/μm)**				
Mean ± SEM	0.092 ± 0.004	0.101 ± 0.004	0.098 ± 0.002	0.096 ± 0.003
Range	0.842-0.107	0.093-0.107	0.093-0.102	0.091-0.105
**PSD Length (μm)**				
**Total ASs**				
Mean ± SEM	0.2160 ± 0.0049	0.2303 ± 0.0098	0.2163 ± 0.0041	0.2160 ± 0.0039
Range	0.205 - 0.237	0.213 - 0.247	0.208 - 0.221	0.205 - 0.224
**Macular ASs**				
Mean ± SEM	0.2202 ± 0.0059	0.2340 ± 0.0110	0.2230 ± 0.0020	0.2225 ± 0.0032
Range	0.208 - 0.247	0.214 - 0.252	0.219 - 0.226	0.214 - 0.230
**Perforated ASs**				
Mean ± SEM	0.1797 ± 0.0058	0.1793 ± 0.0129	0.1750 ± 0.0150	0.1683 ± 0.0082
Range	0.169 - 0.202	0.157 -0.202	0.148 - 0.200	0.149 - 0.189

* $p < 0.05$ and ** $p < 0.01$ vs. EVV group.
n = 6 (EVV); n = 3 (72 h); n = 3 (1 w); n = 4 (4 w) animals
Abbreviations: AS, asymmetric synapses

Supplementary Material

Materials and methods

Behavioral tests

Adjusting stepping test

The adjusting stepping test was performed to monitor the effects of progressive dopaminergic depletion on motor activity *in vivo*. Animals were held by the experimenter with one hand, fixing the hind limbs and slightly raising their hind quarters above the surface of the table. With the other hand, the experimenter fixed one of the upper limbs and the animals were slowly moved approximately 0.9 m sideways across a table over 5 s in both directions. The test was repeated twice for each animal in each session and the average number of adjusting steps in both directions (adduction and abduction) with each forepaw were considered in the analysis. A decrease in the number of steps performed by the animal was considered an adequate measure of bradykinesia.[1,2]

Open field test

Spontaneous locomotor activity and anxiety-like behaviour were assessed with the open field (OF) test. Animals were given one habituation session to explore the open arena (1 m long x 1 m wide x 60 cm high) for 15 min in dim light, and on the same day the OF test was performed. Animals were allowed to explore the arena for 15 min and the activity was video recorded. Using the Ethovision X13 software (Noldus Information Technology), the following parameters were analysed: total distance travelled (cm), total velocity (cm/s), % of the time moving as well as % of time spent in the centre as a measure of anxiety-like behaviour.

Antibodies

Supplementary Table 1. Primary antibodies used for immunohistochemistry and immunofluorescence.

Antigen	Host Species	Species reactivity*	Clone	Isotype	Reference	Producer	Dilution
**DAT**	Goat	H, M, R	Polyclonal	IgG	Sc-1433	Santa Cruz Biotechnology	1:100
**Lamp1**	Rat	H, M, R	Monoclonal (1D4B)	IgG2a	sc-19992	Santa Cruz	1:50
**LC3B**	Rabbit	H, M, R	Polyclonal	IgG	L7543	Sigma Aldrich	1:250
**Rab5**	Goat	H, M, R	Polyclonal	IgG	ABIN144056	Antibodies-online	1:100
**Rab7**	Rabbit	H, R	Polyclonal	IgG	ab77993	Abcam	1:100
**TH**	Mouse	H, M, R	Monoclonal (2/40/15)	IgG2a	MAB5280	Merck Millipore	1:1,000
**α-syn**	Mouse	H	Monoclonal (LB509)	IgG1, kappa	180215	ThermoFischer Scientific	1:500

*We only show the following species: H, human; M, mouse; R, rat.

Supplementary Table 2. Secondary antibodies used for immunohistochemistry and immunofluorescence.

Target	Species of Production	Fluorophore/ Conjugated	Isotype	Reference	Producer	Dilution
**Anti-goat**	Rabbit	Biotinylated	IgG	BA-5000	Vector Laboratories	1:500
**Anti-mouse**	Horse	Biotinylated	IgG	BA-2000	Vector	1:500
**Anti-mouse**	Goat	Alexa Fluor 633*	IgG	A21052	Invitrogen	1:500
**Anti-rabbit**	Goat	Alexa Fluor 488*	IgG	A11034	Invitrogen	1:500
**Anti-rat**	Goat	Alexa Fluor 546*	IgG	A11081	Invitrogen	1:500
**Anti-goat**	Donkey	Alexa Fluor 488*	IgG	A11055	Invitrogen	1:500
**Anti-mouse**	Donkey	Alexa Fluor 647*	IgG	A31571	Invitrogen	1:500
**Anti-rabbit**	Donkey	Alexa Fluor 546*	IgG	A10040	Invitrogen	1:500

Stereological quantification of TH+ neurons in the SNpc

TH^+ immunolabelled neurons were determined by stereology in regularly spaced 50 μm thick sections spanning the entire SNpc and using an Olympus Bx61 motorized microscope (Olympus) equipped with a DP71 digital camera (Olympus) connected to an XYZ stepper (H101BX, PRIOR), driven by CAST Visiopharm software (Visiopharm). The optical fractionator method was employed using an interactive test grid controlled by the software as described previously.[2,3] A total of 7 sections per animal were quantified covering the entire rostrocaudal extent of the SN_{pc}. TH-immunoreactive neuronal bodies were used as counting units and quantified if they were located inside the counting frame or touching the inclusion lines. These parameters were set to reach an error coefficient below 0.10 (Gundersen, m=0) and 0.05 (Gundersen, m=1). Estimated populations for the SN_{pc} of each hemisphere were averaged across all animals for each group and time point.[4,5]

Relative optical density quantification hα-syn immunoreactivity in the SNpc

To quantify the representative surface expression of hα-syn in the SN_{pc}, 3 representative sections of the SN_{pc} were analysed per animal (approx. –5.30 mm, –5.60 mm and –6.00 mm from Bregma according to stereotaxic atlas). Images of all the sections were acquired using a Zeiss Axioimager M1 microscope (Zeiss) with 5x objective (Zeiss), applying the autowhite option and the same acquisition parameters for all sections. Images were converted to 8-bit greyscale images and the SN_{pc} was defined according to the stereotaxic atlas (Paxinos et al., 1985). The relative optical density (ROD) of the grey levels of hα-syn immunoreactivity were obtained with ImageJ software (NIH) according to the following formula: $ROD = log\ (basal\ gray\ level/signal\ gray\ level)$.[6,7] The mean ROD values were averaged across all animals for each group and time point.

TH, DAT and hα-syn immunoreactivity in the striatum

The extent of TH, DAT and hα-syn expression was determined by ROD quantification. For each marker, three striatal sections per animal were analysed (approx. +1.60 mm, +1.00 mm and -0.26 mm from Bregma according to stereotaxic atlas: Paxinos et al., 1985). Images of all the sections were acquired using a Zeiss Axioimager M1 microscope (Zeiss) with a 2.5x scanning objective (Zeiss) using the same acquisition parameters for all sections. ROD values of immunoreactivity for the striatum (Caudate-Putamen -CPu) were obtained using ImageJ software (NIH) by calculating the average grey value of 8-bit grayscale images in the CPu. Non-specific staining was subtracted, calculating the ROD of a small square placed in the corpus callosum of each section. The ROD values of each CPu were averaged across all the animals for each time point.[3]

SWATH-MS

Synaptosomal fractions were homogenized in lysis buffer containing 7 M urea, 2 M thiourea and 50 mM DTT. The homogenates were spun down at 100,000 × g for 1 h at 15 °C and the protein concentration was measured with a Bradford assay (BioRad). A pool of all samples was used as input to generate the sequential window acquisition of all theoretical mass spectra–mass spectrometry (SWATH-MS) assay library. In-gel digestion was employed to increment the proteome coverage. Protein extracts (30 µg) were diluted in Laemmli sample buffer and loaded onto a 0.75 mm thick polyacrylamide gel, with a 4% stacking gel cast over a 12.5% resolving gel. The gel was stained with Coomassie Brilliant Blue and 12 equal slices from the pooled sample were excised from the gel and transferred into 1.5 mL Eppendorf tubes. The proteins were cleaved with trypsin (1:20, w/w: Promega) at 37 °C for 16 h, as described previously [8]. The purification and concentration of peptides was carried out with a C18 Zip Tip Solid Phase Extraction (Millipore) and the peptides recovered from in-gel digestion were reconstituted into a final concentration of 0.5 µg/µl in 2% ACN, 0.5% FA, 97.5% MilliQ-water prior to performing mass spectrometry.

The MS/MS datasets for spectral library generation were acquired on a TripleTOF 5600+ mass spectrometer (Sciex) interfaced to an Eksigent nanoLC ultra 2D pump

system (Sciex) fitted with a 75 μm ID column (Thermo Scientific 0.075 × 250 mm, particle size 3 μm and pore size 100 Å). Before separation, the peptides were concentrated on a C18 precolumn (Thermo Scientific 0.1 × 50 mm, particle size 5 μm and pore size 100 Å). The mobile phases were: buffer A, 100% water 0.1% formic acid (FA); and buffer B, 100% Acetonitrile 0.1% FA. The column gradient was developed from 2% B to 40% B in 120 min and the column was equilibrated in 95% B for 10 min and 2% B for 10 min. Throughout the process, the precolumn was in line with the column and the flow was maintained along the gradient at 300 nl/min. The output of the separation column was directly coupled to the nano-electrospray source. MS1 spectra were collected in the range of 350-1250 m/z for 250 ms and the 35 most intense precursors with charge states of 2 to 5 that exceeded 150 counts per second were selected for fragmentation using rolling collision energy. MS2 spectra were collected in the range of 230–1500 m/z for 100 ms. The precursor ions were dynamically excluded from reselection for 15 s. The database search and processing of the assay library MS/MS data acquisition was performed using AnalystTF 1.7 (Sciex), and the spectra files were processed with the ProteinPilot v5.0 search engine (Sciex) using ParagonTM Algorithm (v.4.0.0.0: [9]) for the database search. To avoid using the same spectral evidence in more than one protein, the proteins identified were grouped based on MS/MS spectra by the Progroup™ Algorithm, regardless of the peptide sequence assigned. The False discovery rate (FDR) was obtained using a non-linear fitting method [10] and the results displayed were those reporting a 1% Global FDR or better.

Individual protein extracts (20 μg) from all experimental groups (6 groups, 5 independent synaptosomal fractions per group) were subjected to in-gel digestion, peptide purification and reconstitution before mass spectrometry analysis as described previously. For SWATH-MS based experiments, the TripleTOF 5600+ instrument was configured as described previously [11]. Using an isolation width of 16 Da (15 Da of optimal ion transmission efficiency and 1 Da for the window overlap), a set of 37 overlapping windows was constructed covering the mass range 450–1000 Da. As such, 2 μl of each sample was loaded onto a trap column (Thermo Scientific 0.1 × 50 mm, particle size 5 μm and pore size 100 Å) and desalted with 0.1% TFA at 2 μl/min for 10 min. The peptides were loaded onto an analytic column (Thermo Scientific 0.075 × 250 mm, particle size 3 μm and pore size 100 Å), equilibrated in 2% acetonitrile 0.1% FA. Peptide elution was carried out with a linear gradient of 2 to 40% B for 120 min at a

flow rate of 300 nl/min. Eluted peptides were infused in the mass spectrometer. The Triple TOF was operated in swath mode, in which a 0.050 s TOF MS scan from 350 to 1250 m/z was performed, followed by 0.080 s product ion scans from 230 to 1800 m/z in the 37 defined windows (3.05 s/cycle). The collision energy was set to an optimum energy for a 2+ ion at the centre of each SWATH block with a 15 eV collision energy spread.

For label-free quantitative data analysis, the resulting ProteinPilot group file from library generation was loaded into PeakView® (v2.1, Sciex) and peaks from SWATH runs were extracted with a peptide confidence threshold of 99% confidence (Unused Score ≥ 1.3) and a FDR lower than 1%. For this, the MS/MS spectra of the assigned peptides were extracted with ProteinPilot and only the proteins that fulfilled the following criteria were validated: (1) peptide mass tolerance lower than 10 ppm; (2) 99% of confidence level in peptide identification; and (3) complete b/y ions series found in the MS/MS spectrum. Only proteins quantified with at least two unique peptides were considered.

Bioinformatics analysis

The identification of significantly enriched structural complexes and biological processes from the deregulated proteins in synaptosomal fractions was performed using Metascape.[12] Cumulative hypergeometric p values and enrichment factors were calculated and used for filtering to generate the different heatmaps after identifying all the statistically enriched terms (structural complex GO/KEEG terms; biological process GO/KEGG terms, canonical pathways, hallmark gene sets). The remaining significant terms were then hierarchically clustered into a tree based on Kappa-statistical similarities among their gene memberships. A 0.3 kappa score was employed as the threshold to cast the tree into term clusters. The term with the best p-value within each cluster was selected as its representative term and displayed in a dendrogram. The heat map cells are coloured by their p values and grey cells indicate the lack of enrichment for that term in the corresponding list. The interactomes of human and rat α-syn were obtained from the curated Biological General Repository for Interaction Datasets (BioGRID: https://thebiogrid.org [13]. The synaptic ontology analysis was performed using the SynGo platform (https://syngoportal.org) [14]. The “brain expressed”

background set was selected that contains 18,035 unique genes in total, of which 1104 overlap with SynGO annotated genes. For each ontology term, a one-sided Fisher exact test was performed to compare differential datasets and the “brain expressed” background set. The result is shown in the “p value” column. To find terms enriched within the entire SynGO ontology, a multiple testing correction using the FDR was applied (q value column).

Mitochondrial respiration parameters

Supplementary Table 3: Mitochondrial Respiration parameters obtained from the OCR data after sequential injection of the modulators of the ETC

Parameter	Equation
**Basal Respiration**	(Last OCR measurement before Oligomycin injection) – (Non-Mitochondrial Respiration)
**ATP Production**	(Last OCR measurement before Oligomycin injection) – (Minimum OCR measurement after Oligomycin injection)
**H^+ (Proton) Leak**	(Minimum OCR measurement after Oligomycin injection) – (Non-Mitochondrial Respiration)
**Maximal Respiration**	(Maximum OCR measurement after FCCP injection) – (Non-Mitochondrial Respiration)
**Spare Respiratory Capacity**	(Maximal Respiration) – (Basal Respiration)
**Coupling Efficiency (%)**	(ATP Production)/ (Basal Respiration) x 100
**Non-mitochondrial Respiration**	Minimum OCR measurement after Rotenone/antimycin A injection

Fractionation of PSD fraction from synaptosomes and western blot

The fractionation of synaptosomes was continued by adding 1% Tritón X-100 and stirring for 30 min at 4ºC. Then, samples were spum down at 150.000 g for 2 h at 4ºC to obtain the Triton insoluble pellets (PSD fraction) that were resuspended in lysis buffer (24 mM HEPES, 150 mM NaCl, 1% Trirón X-100, 5 mM EDTA)[15]. Protein concentrations were determined by BCA Assay (Pierce). Afterward, 5 µg of protein extracts of PSD fraction were loaded on 8% SDS-PAGE followed by western blot using PVDF blotting membranes (GE Healthcare) and iBright image analyzer (ThermoFischer Scientific). Results were quantified using Image J (NIH) under linear conditions. The primary antibodies used were the following: GluR1 (13185, Cell Signalling) and GluR2/3 (500-3004, Abbo Max).

Electron Microscopy Image analysis

Ultrastructural parameters of TH+ fibres

TH^+ fibres were defined as DAB immunostained electrodense structures. All the TH^+ fibres found were manually traced and their density, area, perimeter and area/perimeter ratio were measured. A total of 9965 TH^+ fibres were analysed. In addition, the density, size (area and perimeter) and shape of all the mitochondria found inside the TH^+ fibres was measured by manually tracing clearly identifiable mitochondria. A total of 1631 mitochondria were analysed. Morphological measurements included the following parameters: aspect ratio (AR), computed as [(major axis)/(minor axis)], which reflects the length-to-width ratio; circularity [4π·(surface area/perimeter)] and roundness [4·(surface area)/(π·major axis)], which are two-dimensional indexes of sphericity with values of 1 indicating perfect spheroids; and Feret diameter, which represents the longest distance (µm) of a selected mitochondrion.[16] Furthermore, mitochondrial ultrastructural defects were quantified based on appearance and classified into the following categories: intact mitochondria, with normal appearing cristae; damaged mitochondria, with either swollen or irregular cristae or with crystalline intermembrane inclusions; and degenerating mitochondria, a mitochondrion inside an autophagic vesicle (mitophagy) or a mitochondrion fused with an electroclear structure.[17] Finally, the density and size of electroclear internal structures inside the TH^+ fibres were measured by manually tracing their boundaries. Electroclear structures were defined as a discernible electron-lucent lumen that contrasted with the electrodense DAB immunostaining and resembled autophagic structures as described previously.[18,19]

Ultrastructural parameters of asymmetric synapses

Asymmetric synapses (AS) were defined by a presynaptic terminal containing spherical synaptic vesicles adjacent to an electron-dense PSD and only clearly identifiable AS were quantified. A total of 7802 ASs were analysed and they were classified into two categories according to the shape of their PSD, as described previously [20]: macular synapses, when they contained a continuous PSD; and perforated synapses, when

contained two or more physically discontinuous PSDs. The density and PSD length of both types of AS were measured. In addition, they were further classified as axospinous (synapses contacting on dendritic spines) and axodendritic (synapses contacting on dendritic shafts) synapses based on their postsynaptic targets.[21] Finally, the size and morphological parameters (area, perimeter and area/perimeter ratio) of presynaptic terminals forming ASs, and the mitochondria inside these terminals, were also measured. A total of 2914 clearly identifiable presynaptic terminals forming AS were analysed.

References for the Supplementary Material and Methods

1. Olsson M, Nikkhah G, Bentlage C, Björklund A. Forelimb akinesia in the rat Parkinson model: differential effects of dopamine agonists and nigral transplants as assessed by a new stepping test. *J Neurosci Off J Soc Neurosci*. 1995;15(5 Pt 2):3863-3875.

2. Jiménez-Urbieta H, Gago B, Quiroga-Varela A, et al. Pramipexole-induced impulsivity in mildparkinsonian rats: a model of impulse control disorders in Parkinson's disease. *Neurobiol Aging*. 2019;75:126-135. doi:10.1016/j.neurobiolaging.2018.11.021

3. Rodríguez-Chinchilla T, Quiroga-Varela A, Molinet-Dronda F, et al. [18F]-DPA-714 PET as a specific in vivo marker of early microglial activation in a rat model of progressive dopaminergic degeneration. *Eur J Nucl Med Mol Imaging*. 2020;47(11). doi:10.1007/s00259-020-04772-4

4. GUNDERSEN HJG, JENSEN EB V., KIEU K, NIELSEN J. The efficiency of systematic sampling in stereology - reconsidered. *J Microsc*. 1999;193(3):199-211. doi:10.1046/j.1365-2818.1999.00457.x

5. West MJ. Stereological methods for estimating the total number of neurons and synapses: Issues of precision and bias. *Trends Neurosci*. Published online 1999. doi:10.1016/S0166-2236(98)01362-9

6. Tatulli G, Mitro N, Cannata SM, et al. Intermittent fasting applied in combination with rotenone treatment exacerbates dopamine neurons degeneration in mice. *Front Cell Neurosci*. 2018;12. doi:10.3389/fncel.2018.00004

7. Vermilyea SC, Guthrie S, Hernandez I, Bondarenko V, Emborg ME. α-Synuclein Expression Is Preserved in Substantia Nigra GABAergic Fibers of Young and Aged Neurotoxin-Treated Rhesus Monkeys. *Cell Transplant*. 2019;28(4). doi:10.1177/0963689719835794

8. Shevchenko A, Tomas H, Havliš J, Olsen J V., Mann M. In-gel digestion for mass spectrometric characterization of proteins and proteomes. *Nat Protoc*. 2007;1(6). doi:10.1038/nprot.2006.468

9. Shilov I V., Seymourt SL, Patel AA, et al. The paragon algorithm, a next

generation search engine that uses sequence temperature values sequence temperature values and feature probabilities to identify peptides from tandem mass spectra. *Mol Cell Proteomics*. 2007;6(9). doi:10.1074/mcp.T600050-MCP200

10. Tang WH, Shilov I V., Seymour SL. Nonlinear fitting method for determining local false discovery rates from decoy database searches. *J Proteome Res*. 2008;7(9). doi:10.1021/pr070492f

11. Gillet LC, Navarro P, Tate S, et al. Targeted data extraction of the MS/MS spectra generated by data-independent acquisition: A new concept for consistent and accurate proteome analysis. *Mol Cell Proteomics*. 2012;11(6). doi:10.1074/mcp.O111.016717

12. Zhou Y, Zhou B, Pache L, et al. Metascape provides a biologist-oriented resource for the analysis of systems-level datasets. *Nat Commun*. 2019;10(1). doi:10.1038/s41467-019-09234-6

13. Oughtred R, Stark C, Breitkreutz BJ, et al. The BioGRID interaction database: 2019 update. *Nucleic Acids Res*. 2019;47(D1). doi:10.1093/nar/gky1079

14. Koopmans F, van Nierop P, Andres-Alonso M, et al. SynGO: An Evidence-Based, Expert-Curated Knowledge Base for the Synapse. *Neuron*. 2019;103(2). doi:10.1016/j.neuron.2019.05.002

15. Jurado S, Benoist M, Lario A, Knafo S, Petrok CN, Esteban JA. PTEN is recruited to the postsynaptic terminal for NMDA receptor-dependent long-term depression. *EMBO J*. 2010;29(16). doi:10.1038/emboj.2010.160

16. Picard M, Shirihai OS, Gentil BJ, Burelle Y. Mitochondrial morphology transitions and functions: implications for retrograde signaling? *Am J Physiol Integr Comp Physiol*. 2013;304(6):R393-R406. doi:10.1152/ajpregu.00584.2012

17. Sisková Z, Mahad DJ, Pudney C, et al. Morphological and functional abnormalities in mitochondria associated with synaptic degeneration in prion disease. *Am J Pathol*. 2010;177(3). doi:10.2353/ajpath.2010.091037

18. Jung M, Choi H, Mun JY. The autophagy research in electron microscopy. *Appl Microsc*. 2019;49(1). doi:10.1186/s42649-019-0012-6

19. Martinet W, Timmermans JP, De Meyer GRY. Methods to assess autophagy in situ - Transmission electron microscopy versus immunohistochemistry. In: *Methods in Enzymology*. Vol 543. ; 2014. doi:10.1016/B978-0-12-801329-8.00005-2

20. Hara Y, Park CS, Janssen WGM, Roberts MT, Morrison JH, Rapp PR. Synaptic correlates of memory and menopause in the hippocampal dentate gyrus in rhesus monkeys. *Neurobiol Aging*. 2012;33(2). doi:10.1016/j.neurobiolaging.2010.09.014

21. Montero-Crespo M, Domínguez-Álvaro M, Alonso-Nanclares L, DeFelipe J, Blazquez-Llorca L. Three-dimensional analysis of synaptic organization in the hippocampal CA1 field in Alzheimer’s disease. *Brain*. 2021;144(2). doi:10.1093/brain/awaa406
